# Melittin - the main component of bee venom: a promising therapeutic agent for neuroprotection through keap1/Nrf2/HO-1 pathway activation

**DOI:** 10.1186/s13020-024-01020-x

**Published:** 2024-11-28

**Authors:** Cong Duc Nguyen, Jaehee Yoo, Sang Jun Jeong, Hai-Anh Ha, Ji Hye Yang, Gihyun Lee, Jeong Cheol Shin, Jae-Hong Kim

**Affiliations:** 1 College of Korean Medicine, Dongshin University, Naju, 58245 Republic of Korea; 2 Faculty of Pharmacy, College of Medicine and Pharmacy, Duy Tan University, Da Nang, 550000 Vietnam; 3 Department of Acupuncture and Moxibustion Medicine, Dongshin University Gwangju Korean Medicine Hospital, 141, Wolsan-ro, Nam-gu, Gwangju City 61619, Republic of Korea , 141 Wolsan-Ro Nam-Gu, Gwangju, 61619 Republic of Korea

**Keywords:** Melittin, Bee venom, Neurodegeneration, Antioxidant, Keap1, Nrf2, HO-1

## Abstract

The Nuclear factor erythroid 2–related factor (Nrf2)/ Heme oxygenase-1 (HO-1) pathway, known for its significant role in regulating innate antioxidant defense mechanisms, is increasingly being recognized for its potential in neuroprotection studies. Derived from bee venom, melittin's neuroprotective effects have raised interest. This study confirmed that melittin specificity upregulated the weakened Nrf2/HO-1 signaling in mice brain. Interestingly, we also revealed melittin’s efficient tactic, as it was suggested to first restore redox balance and then gradually stabilized other regulations of the mouse hippocampus. Using a neuro-stress-induced scopolamine model, chromatography and mass spectrometry analysis revealed that melittin crossed the compromised blood–brain barrier and accumulated in the hippocampus, which provided the chance to interact directly to weakened neurons. A wide range of improvements of melittin action were observed from various tests from behavior Morris water maze, Y maze test to immune florescent staining, western blots. As we need to find out what is the focus of melittin action, we conducted a careful observation in mice which showed that: the first signs of changes, in the hippocampus, within 5 h after melittin administration were the restoration of the Nrf2/HO-1 system and suppression of oxidative stress. After this event, from 7 to 12.5 h after administration, a diversity of conditions was all ameliorated: inflammation, apoptosis, neurotrophic factors, cholinergic function, and tissue ATP level. This chain reaction underscores that melittin focus was on redox balance's role, which revived multiple neuronal functions. Evidence of enhancement in the mouse hippocampus led to further exploration with hippocampal cell line HT22 model. Immunofluorescence analysis showed melittin-induced Nrf2 translocation to the nucleus, which would initiating the translation of antioxidant genes like HO-1. Pathway inhibitors pinpointed melittin's direct influence on the Nrf2/HO-1 pathway. 3D docking models and pull-down assays suggested melittin's direct interaction with Keap1, the regulator of the Nrf2/HO-1 pathway. Overall, this study not only highlighted melittin specifically effect on Nrf2/HO-1, thus rebalancing cellular redox, and also showed that this is an effective multi-faceted therapeutic strategy against neurodegeneration.

**Supplementary Information:**

The online version contains supplementary material available at 10.1186/s13020-024-01020-x.

## Introduction

In the realm of neuroscience, addressing neurodegeneration remains a critical objective. Neuroprotection, which involves pharmacological strategies to prevent or mitigate biochemical damage and cellular death in the nervous system, is central to these efforts [1]. Recent studies suggest that neutralizing oxidative stress and restoring cellular redox balance offer a more comprehensive approach to neuroprotection than focusing solely on anti-inflammatory strategies [2, 3]. Genes that regulate antioxidant defenses and detoxification processes play a vital role in maintaining cellular homeostasis and preventing inflammatory triggers [3–5]. Emerging research underscores the role of imbalanced reactive oxygen species (ROS) and excessive electrophiles as primary drivers of neuroinflammation, which leads to neuronal dysfunction.The transcription factor Nrf2 is pivotal in this context, as it regulates the expression of antioxidant genes by binding to the antioxidant response element (ARE) and initiating the transcription of key antioxidants and detoxifying enzymes [6]. Once cellular redox is rebalanced, it facilitates cellular homeostasis and helps prevent an inflammatory chain reaction from occurring [7, 8]. Also, a reduced Nrf2/HO-1 state has been known to be associated with neurodegenerative diseases, and reactivating this pathway is recognized as a promising strategy in neurodegeneration prevention [2, 9–14]. This suggests a key and holistic strategy to utilize Nrf2 activators to reduce oxidative stress as a multi-purpose anti-neurodegeneration approach.

Nrf2 activator compounds, which stimulate antioxidant enzymes such as HO-1 production, emerging as new a-rounded medications strategy [15]. Compounds such as sulforaphane and dimethyl fumarate, both potent Nrf2 activators, have shown considerable promise in clinical trials for reducing oxidative stress and inflammation—key factors in neurodegenerative diseases. Sulforaphane, derived from cruciferous vegetables, has been demonstrated to upregulate Nrf2, thereby enhancing antioxidant and detoxification enzyme expression, which is crucial in preventing neuronal apoptosis and mitigating cognitive decline [16]. Dimethyl fumarate has already been approved as a treatment for relapsing-remitting multiple sclerosis, is also effective in modulating immune responses and reducing neuroinflammation, this protects neurons from oxidative stress and supporting cognitive function [16]. Moreover, Nrf2 activators like bardoxolone and bardoxolone methyl have demonstrated significant antiviral effects, particularly in inhibiting SARS-CoV-2 replication. This suggests that Nrf2 activation could provide a multifaceted therapeutic approach, combining antiviral activity with the cytoprotective effects of the Nrf2 pathway, offering potential benefits in treating COVID-19 and other viral infections [17].

Bee venom, a long-implemented medication in Korean and Chinese traditional medicine, and particularly its main component melittin, has gained increasing attention for its neuroprotective effects [18, 19]. Although previous studies have shown that melittin can restore cellular redox balance via the Nrf2/HO-1 pathway, among many other cellular signaling pathway enhancements, and the precise molecular mechanisms, and the sequence of interactions, that led to the overall therapeutic effects remain unclear [20–22]. Additionally, to our knowledge, in neurodegeneration research, no research has yet investigated whether melittin can upregulate the weaken Nrf2/HO-1 system in an in vivo level, meaning with a neurodegenerative animal model. Thus, clarifying this would be beneficial to explain melittin anti-neurodegeneration characteristics and later clinical applications.

The brain's high oxygen consumption makes it particularly susceptible to oxidative stress. Its significant lipid content further exacerbates this vulnerability, making lipid peroxidation, as indicated by Malondialdehyde (MDA), a hallmark of brain oxidative damage and a key frequently used marker of stress [23–26]. Additionally, glutathione (GSH), the brain's primary antioxidant, which plays a crucial role in neutralizing oxidative stress, with its depletion highlighting the brain's susceptibility to damage [23, 27]. Therefore, MDA and GSH were utilized in this research to indicate brain oxidative stress damage.

Previous studies have demonstrated a wide and dosage-dependent neuroprotective effects of melittin, though they did not fully elucidate the underlying mechanisms [22, 28]. In this research, we selected one dosage of 0.1 mg/kg, administered every other day (within the effective range of previous studies), to study more intensively how melittin impacts the brain, particularly focusing on the hippocampus and its regulatory mechanisms. We utilized the scopolamine model, a widely employed method to simulate Alzheimer-like condition by inducing memory deficits by blocking muscarinic receptors and generating oxidative stress [29–31]. The intraperitoneal injection (I.P.) of scopolamine also allowed us to assess melittin's ability to cross the blood-brain barrier (BBB) without compromising brain integrity, unlike Intracerebroventricular injection of other neuro stress inducers which a needle must penetrate the skull and brain [32].

Our in vivo experiments, coupling with mass spectrometry analysis, provided strong evidence that melittin could accumulate in neurodegenerative mice’s hippocampus tissue, and had the chance to directly exhibit neuroprotective effect onto neurons. Melittin improvement effects were holistic, this compound reactivates the compromised Nrf2/HO-1 pathway among with multiple signalling’s improvement in hippocampus tissue of plagued mice. Next, another second in vivo experiment was set up to screen which improvement among those mentioned was first to take place: This showed Nrf2/HO-1 improvement and cellular redox rebalancing were the first to be detected, which hours later led to downstream improvements in inflammation, apoptosis, neurotrophic factor regulations, cholinergic function, and tissue ATP level. This shifted our focus onto investigate how melittin interact with the Nrf2/HO-1 pathway. We utilized the glutamate-stressed HT22 hippocampal cell model, a potent model in neuro-research [33–37]. This is supported by the fact that in the neurodegenerative mice group, the mentioned mass spectrometry analysis confirmed that melittin could accumulate in the hippocampus and interact directly with neurons. Using pathways inhibitors, this in vitro experiment on Ht22 cell suggested melittin could interact directly to Nrf2/HO-1 system, possibly bypass many intermediates signaling pathways. Further docking and pulldown assay identified melittin direct physical interaction with Keap1, which can lead to Nfr2 liberation in the cytoplasm, and naturally migrate into the nuclear for antioxidant genes expression, such as HO-1.

**Fig. 1:**
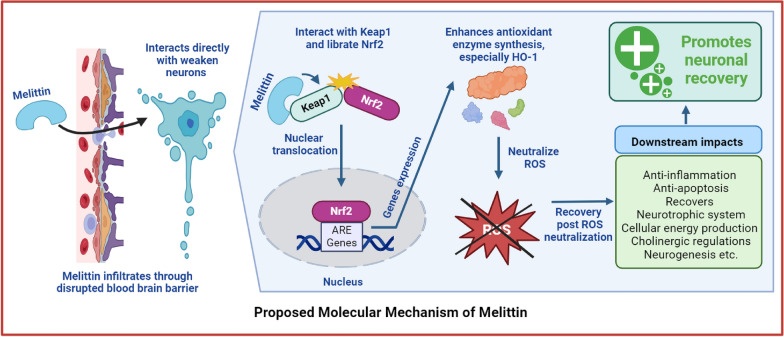
The proposed action of melittin in combating neurodegeneration involves its interaction with the Keap1/Nrf2/HO-1 pathway. Specifically, melittin may interact directly with the Keap1 molecule, which is a crucial step in this process. This interaction leads to the activation of the cellular antioxidant system. Once activated, this system effectively neutralizes harmful reactive oxygen species (ROS) damages, which are known to contribute to neurodegenerative conditions. This holistic mechanism positions melittin as a potential therapeutic agent for neurodegenerative diseases by bolstering the body's natural defense against oxidative

Together, these results suggest that melittin holds promise as a holistic therapeutic agent for conditions characterized by oxidative stress, supported by robust evidence from both in vivo and in vitro studies (Fig. 1).

## Material and method

### Animal and group segregation

One hundred and twenty-two 6-week-old male BALB/c mice were obtained from Samtaco (Gyeonggi-do, Republic of Korea). The mice were housed under controlled conditions, including a 12-hour light-dark cycle at a temperature of 25±3°C and constant humidity. Food and water were provided ad libitum. Each mouse was kept in individual cage. After a 6-day acclimatization period, the mice were randomly divided into groups for two in vivo experiments. Melittin with 97% purity was used (M4171, Sigma-Aldrich, MO, USA). For dosage administration, there was one research which utilized melittin at 0.1 mg/kg twice per week and observed significant effects, and another research with 0.15 mg/kg once every two days; therefore, we picked a dose within this spectrum: 0.1 mg/kg and administered once every 2 days [22, 28].

**Fig. 2:**
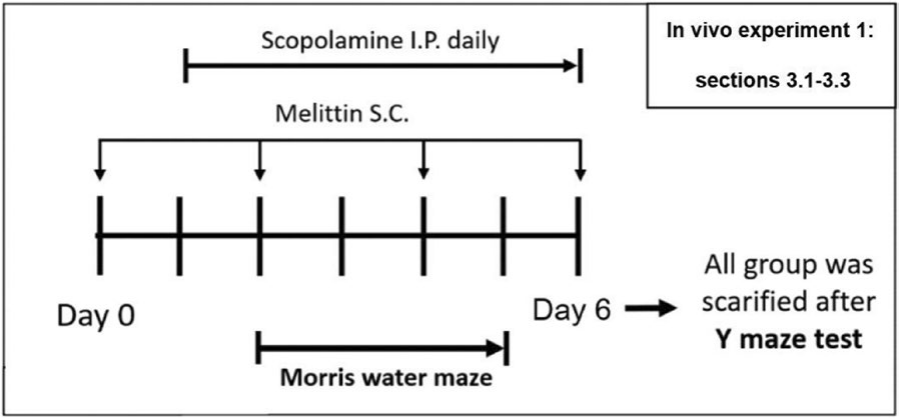
In vivo experiment 1 (results shown in Fig. 3–5): The experimental scheme for melittin effects on behavior amnesia induced by scopolamine. After sacrifice, analysis was carried out for parameters: neurogenesis level,

1. First In Vivo Experiment (Fig. 2): We administered melittin subcutaneously (or sham PSB in other groups) once before scopolamine (6533–68-2, Sigma-Aldrich, MO, USA) administration, this helps identify and exclude mice with adverse reactions, such as reduced food intake (from food leftover calculation), lethargy, or piloerection. This ensures that any negative outcomes observed are due to scopolamine's effects, not from melittin (or sham PBS), thereby maintaining the integrity of the study's findings on neuroprotection. The mice were divided into four groups with eight mice each:
2. Control group: Phosphate-buffered saline (PBS) intraperitoneally (I.P.) and subcutaneously (S.C.)
3. Melittin-only group: PBS I.P. and Melittin 0.1 mg/kg S.C.
4. Scopolamine-only group: Scopolamine 1 mg/kg I.P. and PBS S.C.
5. Scopolamine and Melittin group: Scopolamine 1 mg/kg I.P. and Melittin 0.1 mg/kg S.C.

**Fig. 3:**
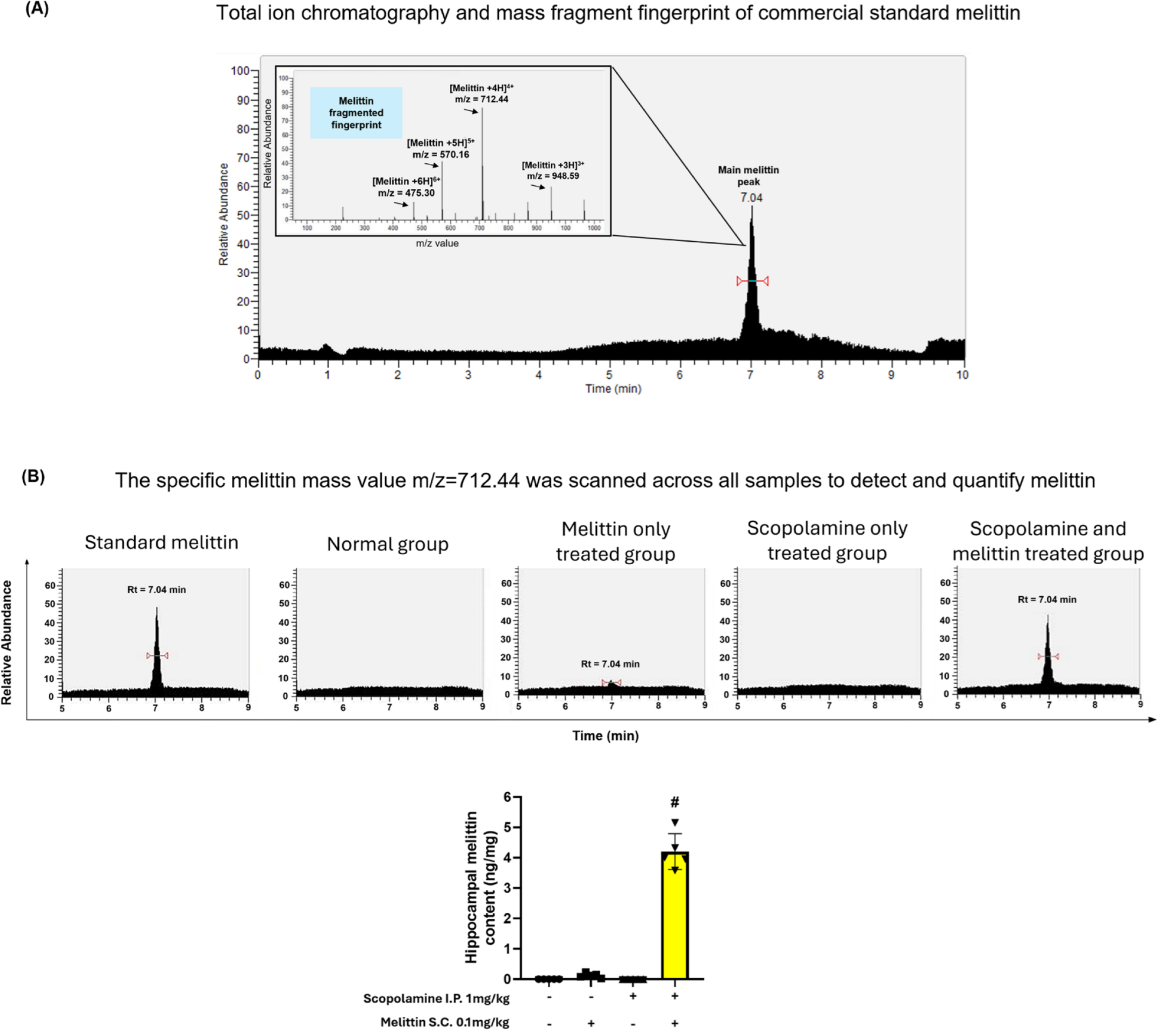
Mass spectrometry analysis provided evidence of melittin passing through disrupted blood brain barrier and accumulated in hippocampus tissue. # p < 0.01 compared with the melittin-only treated group. **A** Total ion chromatography and full mass scan of the commercial standard melittin peak. **B** The value m/z = 712.44 was used to scan across samples to identify and quantify the melittin signal. These discover melittin peaks was check again via there full mass scan to fit with the presented full mass characteristic. Data are presented as mean ± standard deviation. # p < 0.01 compared with the melittin only treated group

**Fig. 4:**
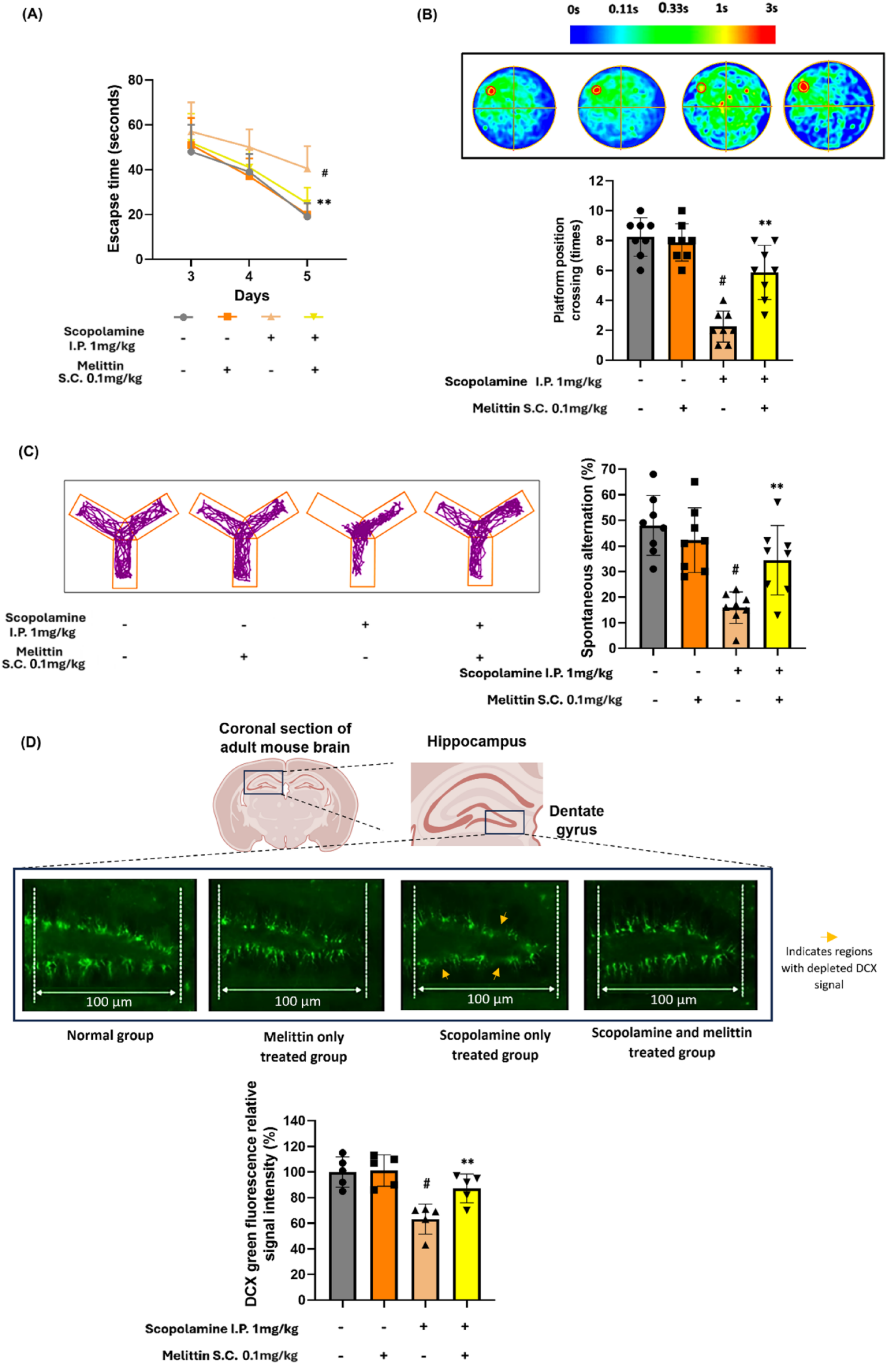
Melittin reversed cognitive impairment in scopolamine-treated mice. **A** Days 3–5, mice were allowed to swim freely for 100 s to find the platform. The escape latency time to target escape was recorded days 7 to 9. **B** The platform was removed, and mice were allowed to swim freely for 120 s. Platform crossing times in the probe test were recorded, the coverage position of where animals swam in each group was used to generate average heatmap of each group. **C** This shows the recorded Y-maze track of one representative animal from each group and spontaneous alternation percentage result. Behavioral score of mice were sharply enhanced by melittin treatment **D** Histochemical analysis revealed that the suppression of neurogenesis by scopolamine was significantly reversed by melittin. Areas marked with orange arrows indicate regions with depleted DCX, representing weakened neurons. The images display the hippocampal dentate gyrus, stained with a DCX antibody. Data are presented as mean ± standard deviation. # p < 0.01 compared with the non-treatment group. * p < 0.05 compared with scopolamine group; ** p < 0.01 compared with scopolamine group. Measurements were carried out triplicated, total animal tested n = 5/group

**Fig. 5:**
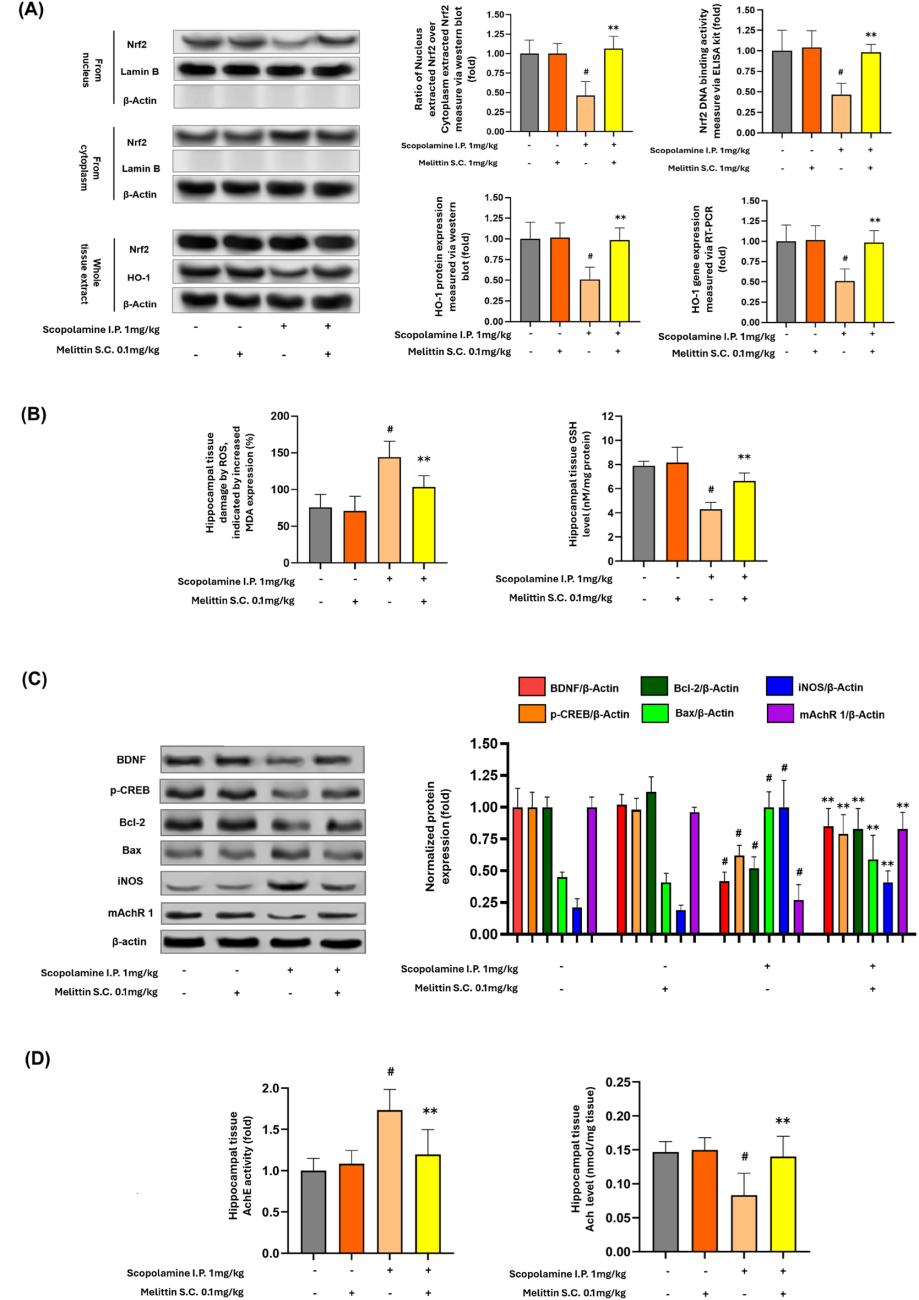
Melittin effects in hippocampus tissue signaling regulations, including reactivation of intrinsic antioxidant barrier, suppress oxidative stress, inflammation, stimulate neurotrophic and cholinergic systems. **A** Westen blot experiment of whole cell, cytoplasm, and nucleus extracts from hippocampus tissue showed melittin enhanced the migration of Nrf2 into the nucleus, which was weaken with scopolamine treatment. Thus, the increased presence of Nrf2 in the nucleus upregulated the important HO-1 antioxidant enzyme to neutralize cellular oxidative stress. Lamin-B was used as the housekeeping marker for nuclear proteins, while β-Actin served as the housekeeping protein for cytoplasmic proteins. No detectable lamin-B was found in the cytoplasm, and no detectable β-Actin was found in the nucleus. This indicates that the extraction process was successful, resulting in a clear separation of nuclear and cytoplasmic proteins. **B** After melittin administration, oxidative stress damage, indicated by MDA lipid peroxidation was suppressed; and GSH level increased which indicated antioxidant capacity recovery. **C** The figure shows Western blot results and quantification of markers for multiple regulation system: neurotrophic BDNF and p-CREB, apoptosis Bcl-2 and Bax, inflammation iNOS, and cholinergic mAChR1 proteins expressions under different treatments. Scopolamine significantly altered the expression of these proteins, while co-treatment with Melittin (0.1 mg/kg) restored their levels towards normal. This indicates Melittin's potential neuroprotective effect in a scopolamine-induced model. **D** Melittin enhanced cholinergic system, we could see a reduction in AchE activity (fold) and Ach concentration enhancement (nmol/mg protein). Data are presented as mean ± standard deviation. # p < 0.01 compared with the non-treatment group; * p < 0.05 compared with Scopolamine group; ** p < 0.01 compared with Scopolamine group. Measurements were carried out triplicated, total animal tested n = 5/group

Melittin was administered days 0, 2, 4, 6; and scopolamine was administered day 1 to 6. These groups underwent treatments, behavioral tests, and were then sacrificed for biochemical analysis (Figs. 3, 4 and 5).

## Second in vivo experiment (Fig. 6 and 7):

**Fig. 6:**
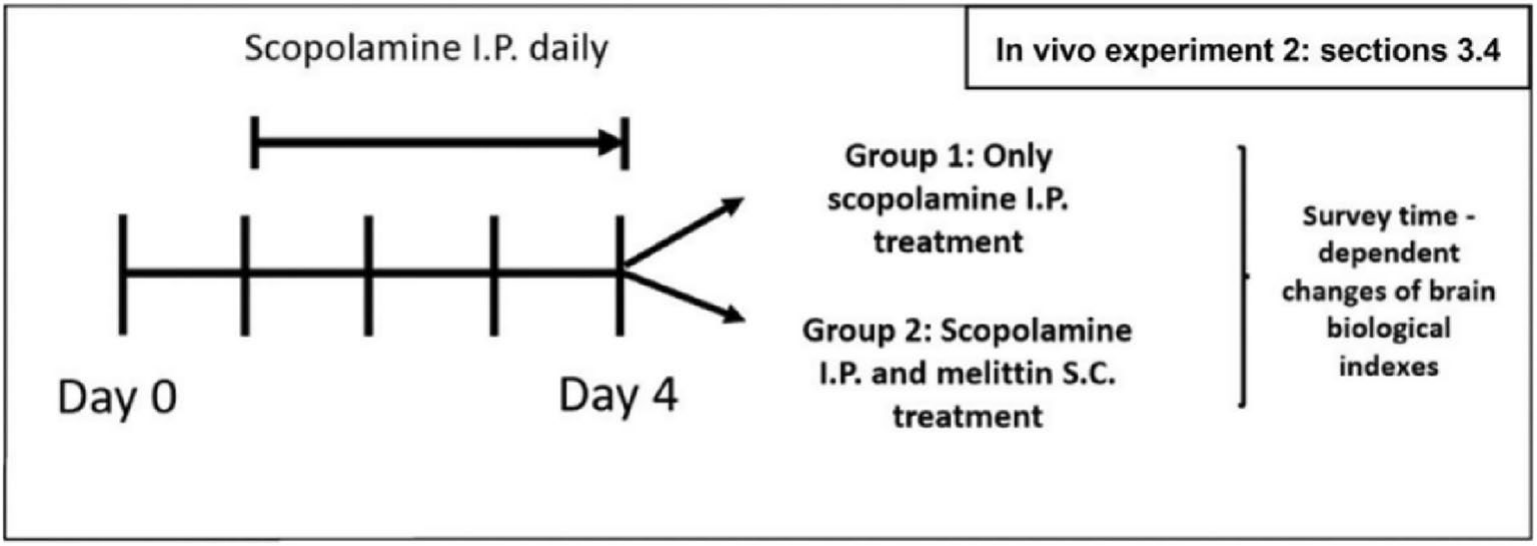
In vivo experiment 2 (results shown in Fig. 7): The experimental for the effect of melittin in a time dependent manner on Nrf2 DNA binding activity; HO-1 gene expression, MDA and GSH levels; iNOS, TNF-α, IL-1β, and IL-6 gene expression; Brain Ach and ATP levels, BDNF and Bcl-2 gene expression. S.C., subcutaneous injection; I.P., Intraperitoneal injection; Scopolamine I.P.: 1 mg/kg; Melittin S.C.: 0.1 mg/kg

**Fig. 7:**
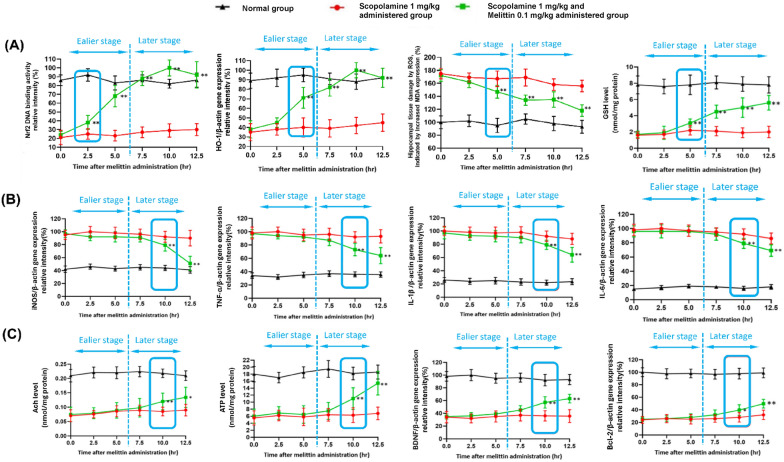
The findings suggest that the foremost notable impacts, happened in the earlier stage (0 to 5 h) after melittin administration were the restoration of Nrf2 DNA binding activity, leading to an increase in HO-1 gene expression to maintain cellular redox equilibrium; This first event was then subsequently followed by the rebalancing of inflammatory, apoptotic, cholinergic, and neurotrophic systems all happened in the later state (7.5 to 12.5 h). **A** Early activation and fortification of antioxidant mechanisms by Melittin: Within just 5 h of administration, a substantial elevation in Nrf2 DNA binding activity was observed. This subsequently triggered a pronounced rise in HO-1 gene expression, resulting in decreased levels of brain tissue MDA and enhanced GSH levels. (**B**) The expression of key inflammation regulators, including iNOS, TNF-α, IL-6, and IL-1β, was assessed. The results indicate that the stabilization of the inflammatory state did not become significant until the 10th hour. (**C**) Enhancement of acetylcholine neurotransmitters, brain ATP content, as well as Brain-derived neurotrophic factors BDNF and Bcl-2 anti-apoptosis gene expression, all exhibited no significant changes until the 10th hour (to the right of the blue dotted line); Data are presented as mean ± standard deviation. #p < 0.01 vs non-treatment group, * p < 0.01 compared with scopolamine only treated group; ** p < 0.001 compared with scopolamine only treated group; Five animals from each group were sacrificed at each time point for the analysis. Measurements were carried out triplicated, total animal tested n = 5/group

This second experiment aimed to evaluate the time-dependent effects of melittin on multiple signaling pathways, including Nrf2 activity, HO-1 gene expression, MDA and Glutathione (GSH) levels, Inducible nitric oxide synthase (iNOS), Tumor necrosis factor-alpha (TNF-α), IL-1β (Interleukin-1 beta), and Interleukin-6 (IL-6) gene expression, as well as hippocampal Acetylcholine (Ach) and Adenosine triphosphate (ATP) levels, BDNF (Brain-derived neurotrophic factor), and Bcl-2 gene expression. On the day of experiment, at 0, 2.5, 5, 7.5, 10, and 12,5 hours after melittin treatment, five mice from each group were sacrificed, and hippocampal samples were analyzed to survey time dependent effect of the treatment (Fig. 7).

The mice were divided into three groups with thirty mice each group:

- Group 1: Received scopolamine 1 mg/kg I.P. from days 1 to 4.
- Group 2: Received scopolamine 1 mg/kg I.P. from days 1 to 4 and additional melittin treatment (0.1 mg/kg S.C.) on the final day.
- Group 3: Received 0.1 ml PBS I.P. from day 1, serving as a reference normal group.

This study was conducted in accordance with the Guide for the Care and Use of Laboratory Animals of the National Research Council (NRC, 1996) and was approved by the Committee of Animal Care and Experimentation of Dongshin University, Korea (DSU2021-01-07). Mice were randomly selected for sacrifice and group assignments using a random number generator (www.calculator.net). Scopolamine was administered I.P. at 4:00 am. Melittin was administered S.C. at 9:00 am at a non-acupoint (Hypochondrium 10 mm above the iliac crest) to avoid interference from acupuncture effects. PBS injection served as the placebo. Behavioral experiments for the assigned group were conducted at 7:00 PM.

### Morris water maze test

Ten hours after the scopolamine injection - 5 hours after melittin injection, the behavior experiment commenced. The Morris water maze was assembled by filling a black circular tank with water (diameter: 120 cm & height: 50 cm) decorated by various graphical indicators on a pole in a fixed position during the whole experiment. Water temperature was maintained at 22±2℃. The tank area was virtually split into four quadrants: southeast, northeast, southwest, and northwest. A white platform with 10 cm diameter and 25 cm height – was placed in the middle of the northwest quadrant. The swimming movement of the mice was evaluated using the Any-Maze software (Stoelting Co., Wood Dale, U.S.A.).

On day 1, an adaptation exercise was accomplished. The animals were permitted to swim freely for 100 s in the tank with the observable platform 1 cm above the water. This was performed three times a day for every mouse; if a mouse was incapable of finding the platform, it was manually led to the position.

From day 2 to day 5. The platform was submerged 1 cm below its surface. The mouse was laid at the center of the northeast quadrant in the first experiment and at the center of the southwest quadrant in the second experiment. The mouse was then allowed to find the platform within 100 s, and if the platform could not be found within 100 s, the mouse was moved to the platform and kept for 10 s. The mouse was then gently placed in a warm water bag and moved back to the cage.

After the above experiment, each mouse was allowed to swim freely for 120s in the tank, however the platform was removed. The swimming patterns were collected to create heatmaps to evaluate each group memory of the platform position[38, 39].

### Y-maze test

On day 6, 10 hours after the scopolamine injection - 5 hours after melittin injection, Y-maze task was executed. The Y-maze is a three-arm maze (40 cm in length, 3 cm in width, and 12 cm high) in which the three arms, made of black polyvinyl plastic, are symmetrically separated at 120°. Mice were initially placed within the same arm, and the arm entry order was recorded over a 5 min period. In this experiment, a spontaneous alternation was defined as entries into all three arms consecutively: ABC, CAB, or BCA, but not BAB, ABA, or CAC. The ANY-maze animal behavior monitoring software (Stoelting Co., Wood Dale, IL, USA) was used to record and determine the results[40].

### Collection of brain hippocampal tissue

The hippocampus was specifically chosen for its importance in the formation and recalling of memory, this organ is also the focus of Alzheimer's studies [41–43]. For in vivo experiment 1 (Figures 2 to 5) mice were sacrifced on the 6th day after the Y maze test. For in vivo experiment 2 (Figures 6 and 7) 5 mice from each group were sacrifced analyzed at each time point.

All mice were anesthetized with isoflurane 3.5% induction for 3 minutes and 1.5–2.0% maintenance, blood was collected from left ventricles, each mouse was carefully perfused with 7 ml of ice-cold saline and 5 ml ice cold 5% paraformaldehyde, subsequently, their brains were collected. The brains were immediately rinsed with physio- logical saline, and the hippocampus were collected. Subsequently, these samples were subjected to further biochemical analysis on the same day, where 5 right hemispheres from 5 different animals of each group were further fixed in ice cold 5% paraformaldehyde again for immuno-histochemistry analysis.

### Extraction for melittin from hippocampal tissue

Hippocampal tissue was homogenized with -20°C cold methanol (0.25 mL per 50 mg tissue), then -20°C cold chloroform (0.25 mL per 50 mg tissue) were added, mixed, and incubated at 2°C for 15 min. Subsequently ice-cold water (0.25 mL per 50 mg tissue) was added, then the sample was mixed and incubated once more. Phase separation was made by centrifuging the mixture at 13,000 rpm for 5 min at 4℃, the upper aqueous phase containing melittin was collected, these were then freeze dried and then solute in 25μL distilled water for Mass spectrometer analysis.

### Mass spectrometry analysis for melittin quantification and keap1 qualification

For melittin quantification: The melittin after being extracted from the brain’s hippocampus was then went through LC−Tandem mass spectrometry for analysis. Establishment of baseline as well as peak spiking and quantitative calculations was closely followed a former publish research [44–46]. The system comprised of an Ultimate 3000 Ultra-high-performance liquid chromatography, and mass detection was accomplished using an LTQ-Orbitrap Velos (Thermo Fisher Scientific, San Jose, CA). The analytical was column Accucore™ C18+ column 1.5 μm particle size, diameter of 2.1 x 100 mm. The flow rate was set at 0.3 mL/min. Sample injection volume was set at 2.0 μL. Two eluent solvents: water (A) and acetonitrile (B) were used with following gradient: 0–3 min: B at 5%; 3–9 min: B to 100%, 9–9.5 min: hold B at 100. The interface was at the voltage of 4.6 kV and the temperature of 270°C, the detection voltage was at 1.97 kV. In the positive ionization mode, mass survey scans were performed in the FT cell with the span of 100 to 1400 m/z. The automatic gain control was 1 x 10^6^ ions. Melittin with 97% purity was used (M4171, Sigma-Aldrich, MO, USA) was utilized to define analysis condition and melittin peak retention time. The main peak in the total ion chromatography chart was evaluated by typical un-fragmented mass spectrometry profile of melittin. This value was then confirmed with result of other studies; subsequently, this specific mass data is the indicator to detect melittin the analyzed samples.

For Keap1 qualification: Following the visualization of suspected Keap1 band at around 60 kDa position, which will be explained later. To avoid noise in mass analysis caused by Coomassie staining, another identical membrane to the one exhibited in figure 10 was used, but it was stained only with Keap1 primary and secondary antibodies. Then, the similar suspected Keap1 band around 60 kDa was marked and excised carefully so it only contains the suspected band, thus increased purity after extraction. The membrane section was incubated in stripping buffer to remove antibodies and staining reagents. Proteins that attached on this small piece of membrane were removed by using eluting solution 50% acetonitrile (ACN) and 0.1% trifluoroacetic acid (TFA), then evaporated to collect only protein in a test tube before trypsin digestion and MALDI analysis. The resulting peptides were purified using Zip-tip C18, then mixed with an α-cyano-4-hydroxycinnamic acid matrix (2.5 mg/ml) containing 50% ACN and 0.1% TFA, and dried on stainless steel targets. MALDI-TOF MS analysis was performed using an AXIMA-TOF2 mass spectrometer in positive ion mode with settings including a 19 kV source voltage, 5 kHz laser frequency, and 15 μJ laser energy. We confirmed the presence of Keap1 by matching significant peaks at m/z 1803.95, 2066.79, and 2135.1 of the commercial standard mice Keap1 (OPCA03207, Aviva Systems Biology Corporation 6370, San Diego, CA USA).

### Doublecortin (DCX) immuno-histochemistry staining

Post-fixed brain hemispheres were incubated in 27% sucrose for another 24 h at 4°C. After being frozen, brain hemispheres were sliced into 30 μm sagittal sections. At room temperature, sections were blocked in with 6% bovine calf serum for blocking, and then in doublecortin primary antibody (1:400, 2 h), rinsed and then incubated with Alexa Fluor 488 secondary antibody (Ex/Em = 490/525 nm, 1:400, 2 h). After two additional rinses slides were covered in Fluoromount™ Aqueous Mounting Medium then topped with glass coverslips and were sent for microscopic imaging. The images of hippocampal dentate gyrus area were photographed using the Invitrogen EVOS FL Auto Imaging System (Thermo Fisher Scientific, Waltham, MA, USA) with a 20× objective, DCX positive cell were counted in a same area and compared between each group.

### Extraction of nuclear and cytosolic proteins for Nrf2 determination

To extract both nuclear and cytosolic Nrf2, we started by homogenizing hippocampus samples using a hypotonic buffer consisting of 10 mM Tris-HCl (pH 7.5), 10 mM NaCl, and 3 mM MgCl2, which allows cellular swelling while maintaining nuclear integrity, and perform this in cold conditions to prevent protein degradation. Then we centrifuged the homogenate at 1,000 x g for 10 minutes at 4°C to pellet the nuclei; retain the supernatant as it contains the cytosolic proteins was stored for western blot analysis. Resuspend the nuclear pellet in a high-salt nuclear extraction buffer containing detergents and protease/phosphatase inhibitors. After a further high-speed centrifugation at 15,000×*g* for 30 minutes at 4 °C, the supernatant containing nuclear proteins was collected.

### Western blot analysis

Hippocampus samples were homogenized in a 50 mM tris aminomethane hydrochloric acid (Tris-HCl, pH 7.4), solution with phosphate inhibitor and protease inhibitor, at approximately 2°C. First, samples were centrifuged at 12,000 rpm, at 4°C for 10 min, a BCA protein analysis kit (ab102536, Abcam, Cambridge, UK) was used to measure total protein content to normalize  across samples. The protein content was mixed with a 25% volume loading buffer and heated at 95°C for 5 min. Then, an amount of 20 μg protein was loaded into wells and electrophoresed via 8% Sodium dodecyl sulfate–polyacrylamide gel electrophoresis (SEMS-PAGE) gel.

Subsequently, separated proteins were transferred onto a polyvinylidene fluoride membrane (PVDF) membrane. Thereafter, 5% non-fat milk was used to block this membrane at room temperature for 1 h and incubated with anti-Lamin B (1:1200, MABS492, MercK KGaA, Darmstadt, Germany), anti- HO-1 (1:500, ab52947, Abcam, Cambridge, UK), anti-BDNF (1:1200,MABN79, MercK KGaA, Darmstadt, Germany), anti- phosphorylated cAMP response element-binding protein (p-CREB) (1:500, 06-519, Sigma-Aldrich, MO, U.S.A.), anti-iNOS (1:1000, ab178945, Abcam, Cam- bridge, UK), anti-mAchR (1:1000, m1M9808, Merck KGaA, Darmstadt, Germany) and anti-β- actin (1:1200, ab8226, Abcam, Cambridge, UK). After initial antibody probing, the membrane was treated with stripping buffer (62.5 mM Tris-HCl, pH 6.8, 2% SEMS, 100 mM β-mercaptoethanol) at 55°C for 30 minutes. The membrane was then washed with TBS-T three times for 10 minutes each. This allowed for re-probing with a new antibody or staining with Coomassie Brilliant Blue to visualize all proteins.

Thereafter, at room temperature, membranes were first rinsed in phosphate- buffered saline with Tween 20 and then incubated with Horseradish peroxidase-conjugated anti-rabbit secondary antibody (1:500, G-21234, Thermofisher, Massachusetts, USA). Another rinse by PBST, then membranes were treated with ECL prime kit (GERPN2236, Sigma-Aldrich, MO, USA). The Amersham™ ImageQuant™ system (Boston, USA) was used to capture the proteins signals, quantitative analysis was carried out via ImageJ (http://imagej.nih.gov/).

### Biochemical assays

The hippocampus was homogenized PBS at 3℃, then centrifuged at 10,000 × g for 10 min at 4℃ with 10% homogenates, the upper layer, known as tissue’s proteins extract, was collected, and stored at -80℃ for biochemical assessment.

Total protein content was measured via a BCA Protein Assay Kit (ab102536, Abcam, Cambridge, UK). Examinations on cholinergic system were carried out via Acetylcholinesterase (AchE) kit (ab138871, Abcam, Cambridge, UK), Ach kit (STA-603, Cell Biolabs, CA, USA), ATP level assay kit (ab83355, Abcam, Cam- bridge, UK), GSH kit (ab239727, Abcam, Cambridge, UK), and Lipid Peroxidation (MDA) Assay Kit (ab118970, Abcam, Cambridge, UK).

For DNA binding assay. After the nucleus proteins were extracted as in the 2.8 section. Assays were carried out using the DNA-binding activity of Nrf2 was valued using the Trans-AM® Nrf2 kit (Catalog Nos. 50296, Active Motif, Carlsbad, CA, USA). Briefly, in the commercial Enzyme-linked immunosorbent assay (ELISA) plate, 15 μg of collected nuclear extract was incubated with the antioxidant response element (ARE) consensus sequence as well as immobilized mutated or wild-type competitor oligonucleotides. The detection for bound Nrf2 was made via anti-Nrf2 primary antibody (1:1000) and Horseradish peroxidase-conjugated secondary antibody (1:1000). This was followed by chromogenic reaction by 3,3',5,5'-Tetramethylbenzidine substrate, and the absorbance was assessed at 450 nm using a plate reader.

#### Quantitative polymerase chain reaction (PCR) assay

From the homogenized hippocampus tissue, total RNA was separated by extraction by Trizol (Thermo Fisher Scientific, Waltham, MA, USA). cDNA was synthesized via the PrimeScript RT kit (Takara Bio Inc., Osaka, Japan) corresponding to the manufacturer guidelines. PCR assay was executed with 1 μL of cDNA and primer at 0.3 μM. The instrument was a LightCycler® 480 System (F. Hoffmann-La Roche Ltd., Basel, Switzer- land) with the reaction medium as the TB Green Premix DimerEraserTM (Takara Bio Inc., Shiga, Japan). Sequent setup: initialization was first for 30 s at 95 °C, then 40 cycles in amplification were made, subsequently denaturation in 5 s at 95 °C, and for annealing and elongation in 30 s at 72 °C. Normalization to those of β-actin gene was made. Primer sequences:

HO-1:

Forward 5′ CCTTCCCGAACATCGACAGCC-3′,

Reverse 5′- GCAGCTCCTCAAACAGCTCAA-3′.

BDNF exon IX:

Forward 5′- GCCTTTGGAGCCTCCTCTAC -3′

Reverse 5′- GCGGCATCCAGGTAATTTT - 3′.

Bcl-2:

Forward 5’- GCCACCTATCTGAATGACCACC -3′

Reverse 5′- AGGAACCAGCGGTTGAAGCGC -3′.

iNOS:

Forward 5’- CACCTTGGAGTTCACCCAGT -3′,

Reverse 5′- ACCACTCGTACTTGGGATGC -3′.

TNF-α:

Forward 5’- GGTGCCTATGTCTCAGCCTCTT -3′

Reverse 5′- GCCATAGAACTGATGAGAGGGAG -3′.

IL-6:

Forward 5’- AGACAGCCACTCACCTCTTCAG -3′,

Reverse 5′- TTCTGCCAGTGCCTCTTTGCTG -3′.

IL-1β:

Forward 5’- AGACAGCCACTCACCTCTTCAG -3′,

Reverse 5′- TTCTGCCAGTGCCTCTTTGCTG -3′.

β-actin:

Forward 5′-CCAGAGCAAGAGAGGTATCC-3′,

Reverse 5′-CTGTGGTGGTGAAGCTGTAG- 3′.

### HT22 mouse hippocampal cell culture and treatment

Mouse hippocampal HT22 cells (SCC129, Sigma-Aldrich, MO, USA) were cultured in Dulbecco's Modified Eagle Medium (DMEM) supplemented with Fetal bovine serum (FBS) (10%) and penicillin–streptomycin (1%) in 5% CO_2_ environment at 37°C. After 5 passages to obtain cell stocks, the cell was prepared for seeding. The cells were cultured in 96-well plates at 1 × 10^4^ cells/well density in 0.1 mL media. Twenty-four hours after seeding, cells were subjected to drugs treatments for each experiment: melittin only, or melittin + glutamate, or melittin + glutamate + inhibitors. After treatments, these cells were incubated for another 12 h and then tested with the WST-8 kit for cell viability determination. Inhibitors were used to hint out which pathway might be the focus of melittin: JNK Inhibitor SP600125, HO-1 synthesis inhibitor SnPP procured from Sigma–Aldrich (St. Louis, MO, USA). The p38 inhibitor SB203580 from InvivoGen (San Diego, CA, USA). The Akt inhibitor MK-2206 from TargetMol (Wellesley Hills, MA, USA).

### Nrf2 nuclear translocation immunohistochemistry of HT22 cell after melittin treatment

After drugs treatments, at 0 hour and 6 hours, cell were fixed with methanol and were allowed to stand overnight with anti-Nrf2 primary antibody (1:500, ab31163, Abcam, Cambridge, UK) at 4 °C, followed by incubation with GFP secondary antibody (A-11122, Thermo Fisher Scientific, Waltham, MA, USA) and DAPI (1:500, D1306, Thermo Fisher Scientific, Waltham, MA, USA) for another 1 h at room temperature. The cells were photographed using the Invitrogen EVOS FL Auto Imaging System (Thermo Fisher Scientific, Waltham, MA, USA) with a 40× objective. Quantitative analysis was carried out via ImageJ (http://imagej.nih.gov/). As Nrf2 nuclear translocation can happens within 10 hour of stimulation[47]. We picked the 6^th^ hour after stimulation to check for this phenomenal.

### Molecular modeling and molecular docking

To assess the binding affinities and interaction patterns of potential inhibitors with the target protein, molecular modeling and docking were performed. Initially, melittin structures were converted from Simplified Molecular Input Line Entry System (SMILES) codes into three-dimensional (3D) structures using Open Babel, an open-source tool designed for chemical data manipulation. Then, molecular docking was executed using AutoDock Vina, a recognized software for this purpose[48, 49]. The procedure of performing docking was conducted as previously described[50]. The protein model for docking was sourced from the RCSB Protein Data Bank (PDB), specifically using the PDB code **7OFE** to represent the target protein. AutoDock Vina's parameters were set to probe for optimal docking poses within a grid box centered at coordinates (-43.16, 21.16, -11.37) and sized 38.00 Å x 38.00 Å x 38.00 Å level of 128, ensuring a thorough exploration of the conformational space. Post-docking, the results were visualized and analyzed using Chimera and Discovery Studio Visualizer for highlighting hydrogen bonds, hydrophobic contacts, and the fit of ligand within the binding site on the protein.

#### Pull down assay

The pull-down assay was conducted by attaching melittin to Epoxy-activated Sepharose 6B beads (obtained from Sigma, St. Louis, Missouri, USA). Initially, 1 mg of melittin was dissolved in 1 mL of a coupling buffer consisting of 0.1 M NaHCO_3_ and 0.5 M NaCl at a pH of 11. The Sepharose 6B beads were first allowed to swell and were then cleansed with 1 mM HCl using a sintered glass filter. Subsequent washes were performed using the same coupling buffer. The beads were then combined with the melittin-infused coupling buffer and incubated at 4 °C overnight. To block any non-specific binding sites, 1 M ethanolamine was used at 4 °C overnight. The beads, now conjugated with melittin, underwent a series of washes with buffers alternating in pH—the first buffer containing 0.1 M acetate and 0.5 M NaCl at pH 4, and the second containing 0.1 M Tris-HCl and 0.5 M NaCl at pH 8. Post-washing, the beads were stabilized in a binding buffer containing 0.05 M Tris-HCl and 0.15 M NaCl at pH 7.5. Control beads without melittin were prepared using the same procedure. For the assay, HT22 cell lysates were prepared using PRO-PREBP lysis buffer and combined with either the melittin-conjugated Sepharose 6B or the control beads, followed by an overnight incubation at 4 °C. Afterward, the beads were thoroughly washed three times with TBST and the proteins bound to the beads were eluted using SEMS-loading buffer. These proteins were then separated using SEMS-PAGE and analyzed via immunoblotting, employing antibodies specific to Keap1 with dilution 1:800 (A80790, Antibodies, Cambridge, UK), or later with Coomassie Brilliant Blue staining (6104-58-1, Sigma-Aldrich, MO, USA).

#### Statistical analysis

SPSS version 18.0 was used for statistical analysis, and data are expressed as mean ± standard deviation (SD). SEM was chosen to provide an estimate of the precision of the sample mean as an estimate of the true population mean. In quantitative measurements, data were analyzed using two-way ANOVA followed by Tukey's multiple comparison test. Differences between results were considered if the significant with p < 0.05.

## Results

Nrf2 DNA binding activity and HO-1 expression, MDA and GSH index; BDNF, p-CREB, Bcl-2, Bax, iNOS, and mAchR 1 protein expressions; AchE and Ach levels.

### Chemical analysis proved melittin passing through disrupted BBB and accumulated within *hippocampus* tissue:

We first checked if melittin could accumulate in the mice brain or not, this is important later on to explain melittin directly or indirectly affects the brain tissues. The hippocampus was specifically chosen for its importance in the formation and recalling of memory, this brain organ is also the focus of Alzheimer's studies [41–43].

From the Total Ion chromatogram of commercial standard melittin (with a high purity of 98%), we detected a main peak and confirmed to be melittin. The peak tips’ retention time proximity 7.04 min. The regression equation was y = 63.287x—1101.8 (R^2^ = 0.999) and was applied to calculate the level of melittin in samples (Fig. 3A). Specific melittin mass pattern was based on signals broken down from this main peak: [M + 3H]^3+^ m/z = 948.59 (Approximately 1/3 of melittin molecular weight), [M + 4H]^4+^ m/z = 712.44 (Approximately 1/4 of melittin molecular weight), Melittin [M + 5H]^5+^ m/z = 570.16 (Approximately 1/5 of melittin molecular weight), and Melittin [M + 6H]^6+^ m/z = 475.30 (Approximately 1/6 of melittin molecular weight). Within the mass spectrometer chambers, each melittin molecules coupled with several H+ ions, resulting in the multiple m/z values detected. When scanning through all sample’s chromatograms at the m/z = 712.44 value, we pinpointed the peak with the retention time nearest to 7.04 min. This allowed us to detect and quantify melittin.

As a result, from all mice groups, we detected melittin only in hippocampus of mice co-treated with scopolamine and melittin. In the normal group as well as scopolamine only treated groups there was no detectable amount of melittin near the suspected retention time of 7.04 min (Fig. 3B).

In scopolamine and melittin co-treated group, there was a significant amount of melittin detected in hippocampus tissue. As previous research mentioned, the administration of scopolamine can induce stress in the brain and clearly disrupt the BBB [51–53]. This creates an opportunity for large molecules such as melittin to enter the brain tissue such as hippocampus, and produce a direct effect on the hippocampal neurons. Such change is BBB selectivity was reported before: when the BBB is weakened, even CD4 cells can enter into brain tissues, typically, the size of these cells restricts their entry through an intact blood-brain barrier (BBB) [54]. This is the first study to present evidence that melittin can accumulate in hippocampal tissue of neurodegenerative brains. (Fig. 3B). The topic is explored in greater detail in the Discussion section.

We also found weak melittin signals in the melittin only treated group samples. We explain this as: even though all mice were carefully perfused, there could still be a trace amount of melittin in a very small blood volume left, despite after cleaning by perfusion (Fig. 3B).

### Melittin exhibited cognitive protective effect against scopolamine induced amnesia and recover in *hippocampus* neurons neurogenesis:

The flowing first in vivo experiment was about if melittin could enhance cognitive function of scopolamine neuro stress induced mice. Cognitive function encompasses mental processes such as thinking, knowing, remembering, judging, and problem-solving and was tested in this experiment via Morri water maze and Y maze. In the water maze experiment assessing mice's long-term learning memory, notable findings emerged. Initially, scopolamine hindered cognitive function as training progressed, but on days 7-9, the scopolamine-treated group showed minor improvement, reducing latency from above 60 to around 50 seconds. Adding melittin treatment to scopolamine treated mice significantly reduced escape time, when compared to scopolamine only treated group (p<0.01), nearly matching those without scopolamine. The normal and melittin-only groups showed little difference (Fig. 4A). In the probe test, scopolamine impaired mouse memory of the platform's location (p<0.01), while melittin treatment improved their performance, as seen in the heatmap, and increased platform crossing times (p<0.01). Both the normal and melittin-only groups had similar high crossing times as they were the fastest to learn (Fig. 4B).

As the result of observing spontaneous alternation (%) in Y maze test which indicated short term memory ability, it was confirmed that scopolamine significantly decreased the spontaneous alternation index (p<0.01), whereas melittin treatment significantly enhanced this suppression (p<0.01) [55]. There was also no significant difference between the normal and melittin only treated group (Fig. 4C).

The brain's hippocampal physiology is closely tied to the neuroprocessing abilities of mice. One critical aspect of hippocampal function, the status of neuronal genesis in the dentate gyrus, is commonly studied due to its vital role in the development, recollection, and assessment of episodic memory. Brain sections were stained using the DCX antibody, a key marker for assessing neuronal neurogenesis state as it's expressed by developing neurons [41, 42, 56]. The results vividly illustrated the damage inflicted by scopolamine at an anatomical level. Neurogenesis involves the growth and development of nervous tissue, particularly the formation of new neurons in the brain. In this case we monitor via the expression of Doublecortin-positive neurons in hippocampus [56]. In the same region, scopolamine treatment decreased the number of DCX-positive neurons by approximately 60% compared to the normal group (p<0.01). However, the introduction of melittin significantly increased this count up to 80~90% (p<0.01), confirming the neuroprotective effect of this drug against scopolamine-induced stress. Importantly, the group treated exclusively with melittin exhibited no significant difference from the normal group (Fig. 4D). To further elucidate the effect of melittin on the hippocampus, we conducted more closely tests with the HT22 mouse hippocampal neurons in later in vitro parts.

### Melittin showed wide-ranging neuroprotection effects in the examined hippocampus tissue: upregulation of antioxidant defenses and recovery of neurotrophic, inflammatory, and cholinergic functions in hippocampus tissue

After behavior experiment, hippocampus of mice was collected to study multiple aspect of biological to explore how melittin performed its’ neuroprotective effect, as this organ is known to be important to cognitive function [41, 42]. In prior renal-disorder-related studies, it was documented that melittin played a role in modulating the nuclear translocation of the nuclear factor erythroid 2-like 2 (Nrf2), a pivotal transcription factor responsible for upregulating the expression of important antioxidant genes such as heme oxygenase-1 (HO-1) [20]. This intrinsic antioxidant barrier is important and is the cornerstone of combating oxidative stress and slowdown neurodegeneration^57^.

Despite that, this study is the first report to demonstrate that melittin could indeed up regulated Nrf2/HO-1 system within animal brain’s tissue: In the hippocampus samples of scopolamine only treated mice, we could observe a collapse of the Nrf2/HO-1 system, as the nucleus Nrf2 level, its DNA binding activity and the HO-1 expression were only haft of that when compared to the normal group (p<0.01). When we administered melittin to recuse this situation, this treatment induced Nrf2 nuclear translocation (figured by an increase in nucleus and reduction in cytoplasm Nrf2), which led to the recovery of Nrf2 DNA binding activity and subsequently raising HO-1 production (p<0.01) (Fig. 5A).

As the normal state the Nrf2 protein is mostly kept in the cytosolic. In neurodegenerative mice, the Nrf2 signaling is inactive due to prolonged stress condition, thus led to the reduction of Nrf2 nuclear/ Nrf2 cytosolic ratio; the whole cell Nrf2 level might not change much, but they are inactive [9, 58]. The key is under melittin stimulation to group that suffered from scopolamine-induced stress, Nrf2 from cytosolic translocated into the nuclear, which led to increase in Nrf2 nuclear/ Nrf2 cytosolic.

As a result of the increased production of antioxidative enzymes such as HO-1, oxidative stress (ROS) damage, represented via the explained MDA indicator, was suppressed in the melittin and scopolamine co-treated group compared to the group treated with scopolamine alone(p<0.01). Glutathione (GSH) is crucial in the oxidative stress defense system as it acts as a primary antioxidant, neutralizing reactive oxygen species (ROS) and repairing oxidative damage in cells. By acting as a natural buffer against ROS, GSH helps maintain cellular integrity and function. Its levels serve as a key indicator of tissue recovery from oxidative stress, with higher GSH concentrations reflecting improved cellular health and restoration of normal function in damaged tissues [59] (Fig. 5B).

After proofs of cellular redox re-balancing, we screened a diverse range of pathways to see if other cellular regulations also improved by melittin: BDNF and p-CREB are crucial for neuronal survival and plasticity, and their reduced levels imply impaired neuronal health [60]. Muscarinic acetylcholine receptor (mAChR1) is essential for cognitive function, and its decreased expression suggests compromised neurotransmission [61]. iNOS was chosen as an inflammation marker due to its role in producing nitric oxide, leading to oxidative stresss [62, 63]. Bcl-2 promotes cell survival, and its reduction indicates increased cell death, while Bax promotes apoptosis, and its increase signifies heightened apoptotic activity [64, 65].

With scopolamine-induced stress alone, we observed significantly decreased expression of key neuronal signaling proteins, including neurotrophic factors BDNF and p-CREB, and the neurotransmitter receptor protein mAChR1, indicating reduced both impaired neurotrophic support and cholinergic function (p<0.01). Scopolamine also increased inflammation, as evidenced by elevated iNOS protein levels, and promoted apoptosis by decreasing the anti-apoptotic protein Bcl-2 and increasing the pro-apoptotic protein Bax.

When melittin was administered to these dysregulated mice, BDNF and p-CREB levels increased by approximately twofold, and mAChR1 levels nearly doubled, indicating recovery of neurotrophic support and cholinergic function (p<0.01). Melittin also reduced iNOS levels by about half, reflecting decreased inflammation, and normalized apoptotic signaling by increasing Bcl-2 levels by nearly 50% and decreasing Bax levels by about a third, demonstrating an overall neuroprotective effect (p<0.01) (Fig. 5C).

For impact on the neurotransmitter system, an assessment of acetylcholinesterase activity and acetylcholine concentration in brain tissue revealed significant differences between the scopolamine group and the non-treatment group. Notably, the scopolamine only treated group showed an increased in AchE activity and reduction if Ach levels (p<0.01) which is a hallmark of neurodegeneration and could explain the decrease of memory learning ability [66–68].  Compared to scopolamine treated group, melittin and scopolamine co-treated group had indeed reduced the AchE level, as well as increase Ach content in hippocampus tissue(p<0.01).

These findings suggested that melittin effectiveness is comprehensive across multiple aspects of neuro-recovery.

Besides this, the melittin only treated group exhibited little alternations from the normal group.

### Melittin enhance HO-1 gene expression as in the initially stage of action, prior to inflammation and neurotrophic factor response:

In this research, a second, more in depth, animal experiment was conducted (Figs. 6, 7). The positive effect of melittin treatment was holistic across a variety of aspects as presented above. Previous result until now also showed melittin enhanced wide aspect of neuro-protective regulations [22, 28]. To explain what the focus of melittin was, there was a need to examine which direction of improvement might be the initial target. We argued that just a single first administration onto a neurodegenerative situation is sufficient to explore the course of action in a time-dependent manner, providing a foundational understanding of its initial effects. Therefore, we performed a wide range of experiments periodically on mice after being treated with and/or melittin (Fig. 7)**.** PCR experiments were conducted because Gene transcriptions is highly sensitive and can reliably pinpoint which gene is being interacted by melittin effects in a timely prioritized manner [69–72].

The method of using Nrf2 DNA binding activity and HO-1 gene expression had been validated to align with Nrf2 nuclear translocation and HO-1 protein expression in previous section above, therefore these was applied to monitor in this experiment in huge quantity.

The results were surprising. After administering scopolamine, both control and treated groups exhibited a reduction in nucleus Nrf2 DNA binding activity (which were shown to go hand-in-hand with the rate of Nrf2 nuclear translocation, explained in section 3.3 above), decreased HO-1 gene expression, elevated hippocampal ROS, and lower GSH levels. However, when treated with melittin, significant changes in these antioxidant markers were observed as early as the 2.5th to 5th hour (p<0.01) (Fig. 7A). This was notably earlier than the improvements in inflammation parameters, which only became significant around the 10th hour: iNOS, TNF-α, IL-1β, and IL-6 gene expression (p<0.01) (Fig. 7B). Additionally, enhancements in cholinergic parameters like ACh levels, brain neurotrophic factor BDNF and anti-apoptosis Bcl-2 gene expressions, brain ATP levels.

For the first time in our knowledge, a study has revealed that melittin not only upregulates the weakened Nrf2/HO-1 pathway in animals at a neurodegenerative stage but also provides compelling evidence that this pathway is the initial focus of melittin's action, among many other aspects that was improved by melittin after administration. This discovery opens new avenues for targeting neurodegenerative diseases and highlights the profound potential of melittin in therapeutic interventions.

### In vitro experiments provide evidence of melittin's initial and directly activation of the Nrf2/HO-1 pathway, and ignore multiple inflammation and apoptosis related - ERK, JNK, p38, Akt signaling pathways

After in vivo experiments on the mouse hippocampus demonstrated positive results, we conducted this further in-depth in vitro study using the HT22 mouse hippocampal cell line. This was supported by the fact that mass spectrometry analysis confirmed that melittin could accumulate in the hippocampus of plagued mice, and thus had the chance to interact directly with neurons. We aimed to further investigate the mechanisms through which melittin exerts its effects. Glutamate negatively affects HT22 cells by causing oxidative stress, mitochondrial dysfunction, calcium overload, and apoptosis. This widely used in vitro model mimics brain stress, and replicates mechanisms observed in psychiatric and neurodegenerative diseases, making it valuable for studying stress-induced neuronal damage and excitotoxicity [33–37].

In this section, we delved into the intriguing mechanisms by which melittin actively interacts with the Nrf2/HO-1 pathway to exert a profound neuroprotective effect. This neuroprotection is of great interest due to its potential implications for the treatment of neurological disorders and the understanding of cellular stress responses.

**Fig. 8:**
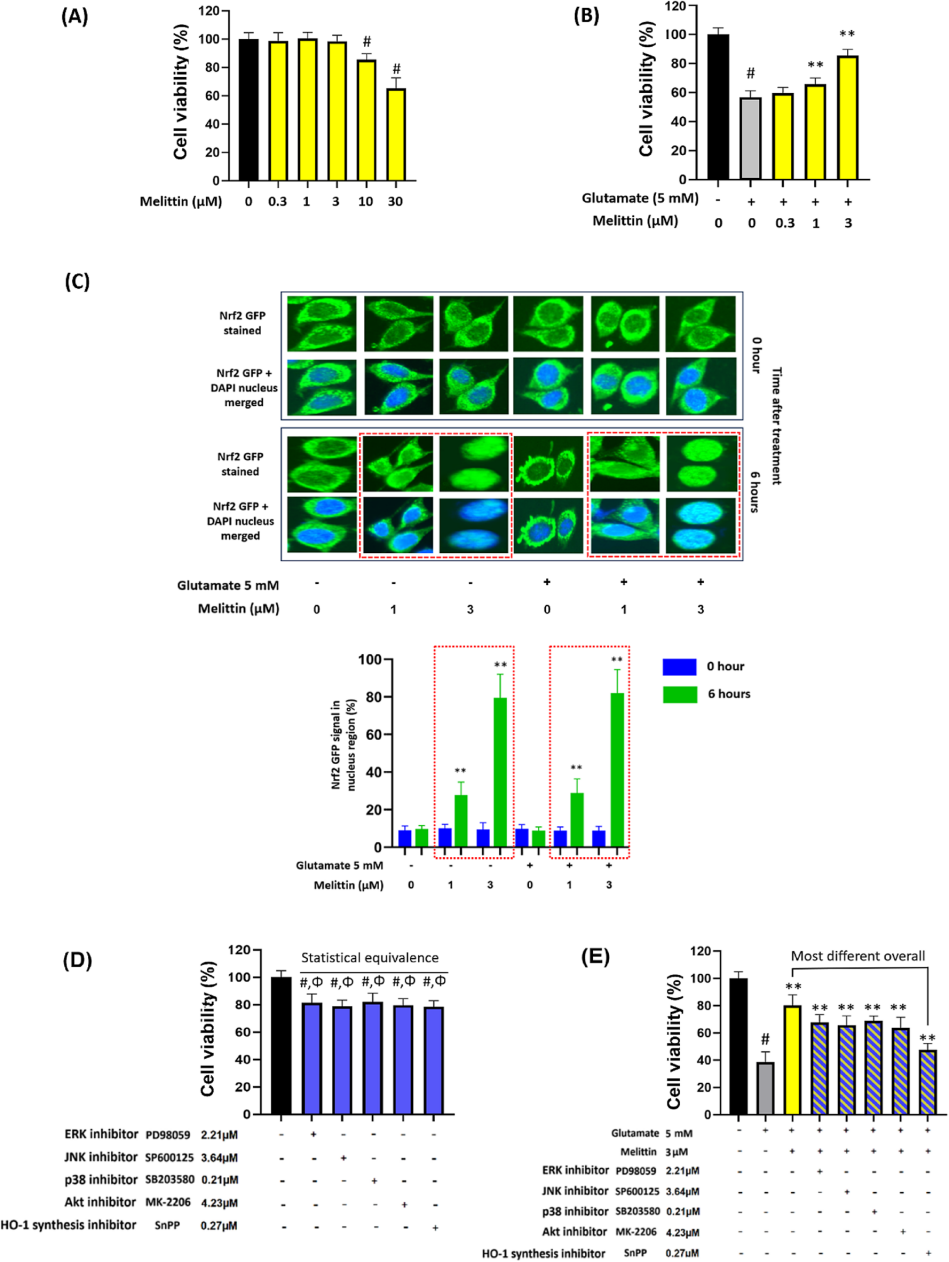
In vitro experiment with mouse hippocampal HT22 proposed that melittin directly engages with the Nrf2/HO-1 pathway to elicit a neuroprotective effect. **A** Screening for the optimal concentration of bee venom revealed that 3 μM melittin was the highest concentration that did not compromise HT22 cell viability 12 h after treatment. **B** Melittin exhibited a dose-dependent neuroprotective effect in the range of 0.3–3 μM against glutamate-induced stress, results measured 12 h after treatment. **C** Fluorescent staining revealed Nrf2 translocation into the nucleus following melittin treatment, with a significant nuclear Nrf2 signal at 1 and 3 μM concentrations. In the red-dotted squares, green, fluorescent Nrf2 signals overlap with the blue neuron nucleus. Higher melittin concentrations increase Nrf2 accumulation in the nucleus, indicating melittin promotes Nrf2 nuclear translocation in a concentration-dependent manner.4 **D**, **E** To elucidate the pathway most closely associated with melittin activity, various inhibitors targeting ERK, JNK, p38, Akt, and HO-1 synthesis were employed. **D** Inhibitor concentrations that inhibited approximately 80% of HT22 cell viability were identified. **E** Co-treatment of these concentrations with melittin and glutamate revealed that the HO-1 synthesis inhibitor exhibited the most pronounced reversal of melittin's positive effects. Data are presented as mean ± standard deviation. # *p* < 0.01 vs. non-treatment group; * *p* < 0.05 and ** *p* < 0.01 vs. glutamate only treatment group; p > 0.05 Statistical equivalence. Measurements were carried out triplicated

First, to identify the ideal concentration of melittin. The objective was to determine the highest melittin concentration that did not compromise the viability of HT22 cells, a crucial step to ensure the safety of this potent compound. It was found that 3 μM of melittin stood as the threshold concentration, effectively preserving the viability of HT22 cells (p<0.01) (Fig. 8A).

Building upon this initial screening, we explored the neuroprotective potential of melittin in a dose-dependent manner. Concentrations ranging from 0.3 μM to 3 μM were tested against glutamate-induced stress, a common stress inducer for HT22 cell line to mimic a neuro-degenerative condition in Alzheimer brains[33–35]. Our results revealed a dose-dependent neuroprotection, with higher melittin concentrations yielding more robust protective effects, the cell availability increased from about 50% up to 80% when treated with melittin (p<0.01) (Fig. 8B).

The pivotal role of the Nrf2/HO-1 pathway in cellular defense against oxidative stress is well-established. When this system is activated Nrf2 is detached from Keap1 in the cytosolic and move into the nucleus, attach to the ARE gene region to activate the transcription of antioxidant genes especially HO-1 [15, 73]. To gain insights into the cellular processes involved, we conducted fluorescent staining experiments. These experiments illuminated the dynamic translocation of Nrf2 into the nucleus following melittin treatment. Significantly, Nrf2 translocation was observed at concentrations of 1 μM and 3 μM of melittin, highlighting the engagement of this pathway in melittin-induced: Groups treated with melittin (despite treatment with glutamate) showed in the 6^th^ hour after treatment, an increase in green florescent signal that overlap the nucleus (p<0.01) (which is stained in blue DAPI), especially the groups with 3 μM melittin exhibited very Nrf2 nuclear translocation that most of Nrf2 in the cytoplasm disappeared and almost all concentrated into the oval-shaped nucleus(p<0.01) (Fig. 8C). Nrf2 nuclear translocation, checking via immune florescence staining is know as a method to visually confirm Nrf2 activator ability of a drug, and is implicated in previous research [74, 75].

To find out which pathway or protein a drug targets, it is common to use inhibitors that suppress specific cellular defense responses. These inhibitors help identify the drug's target because when the right inhibitor is used with melittin, the pathway that the drug activates would now be slowed down, thus the drug healing effect now has little use because the pathway that the drug works on has now been suppressed [37, 58].

Melittin can activate antioxidant defenses via directly upregulation of the Keap1/Nrf2/HO-1 pathway. Or, indirectly via anti-inflammatory and anti-apoptosis responses, which in terms are closely modulated by critical proteins like ERK, JNK, p38, and Akt. Following this direction, we applied inhibitors of ERK, JNK, p38, Akt, and HO-1 synthesis proteins with their respectively concentrations. These inhibitors were used in previous research to determine target of interaction for Nrf2/HO-1 activation [37, 58].

We picked these concentrations, after screening with various dosages before, as they influenced similar cell availability of around 80% (statistically equivalent in comparison). This concentration selection is crucial to achieving consistent and comparable cell growth performance, serving as a relative indicator of similar HT22 cell health conditions in each group, which provides a baseline for subsequent melittin intervention (Fig. 8D).

After introducing the above inhibitor with melittin, the treatment of all inhibitors reduced the drug’s protection. It was feasible since all the pathways broadly contribute to cellular recovery and every pathway that was slowed down could hinder melittin protection effect somehow. However, the inhibitor that reduced the most this protection effect was HO-1 synthesis inhibitor, which was much more significant than other inhibitors. This implied that the most relevance to melittin direct activity might be a component in the Keap1-Nrf2-HO-1 pathway itself (Fig. 8E).

### Docking affinity and stability of melittin on Keap1: potential impacts on Keap1/Nrf2 complex formation:

The above in vivo (Fig. 5A) and in vitro (Fig. 8C) experiments all showed an increase translocation of Nrf2 from the cytoplasm into the nucleus, and in vitro experiments with pathway inhibitors suggested that melittin did this while possibly bypass many intermediates signaling pathways. Typically, Nrf2 is held hostage within the cytoplasm by the Keap1/Nrf2 complex. If melittin perform a direct interaction with the complex, it can distort this complex’s stability via an attachment to the Keap1 molecule, then, the intact Nrf2 can have a chance to be liberated from the complex and allow it to move into the nucleus, where Nrf2 has a natural affinity with the ARE gene promotion area on the DNA, and acts as the main promoter of important antioxidant gene expression such as HO-1 [73, 76–79].

**Fig. 9:**
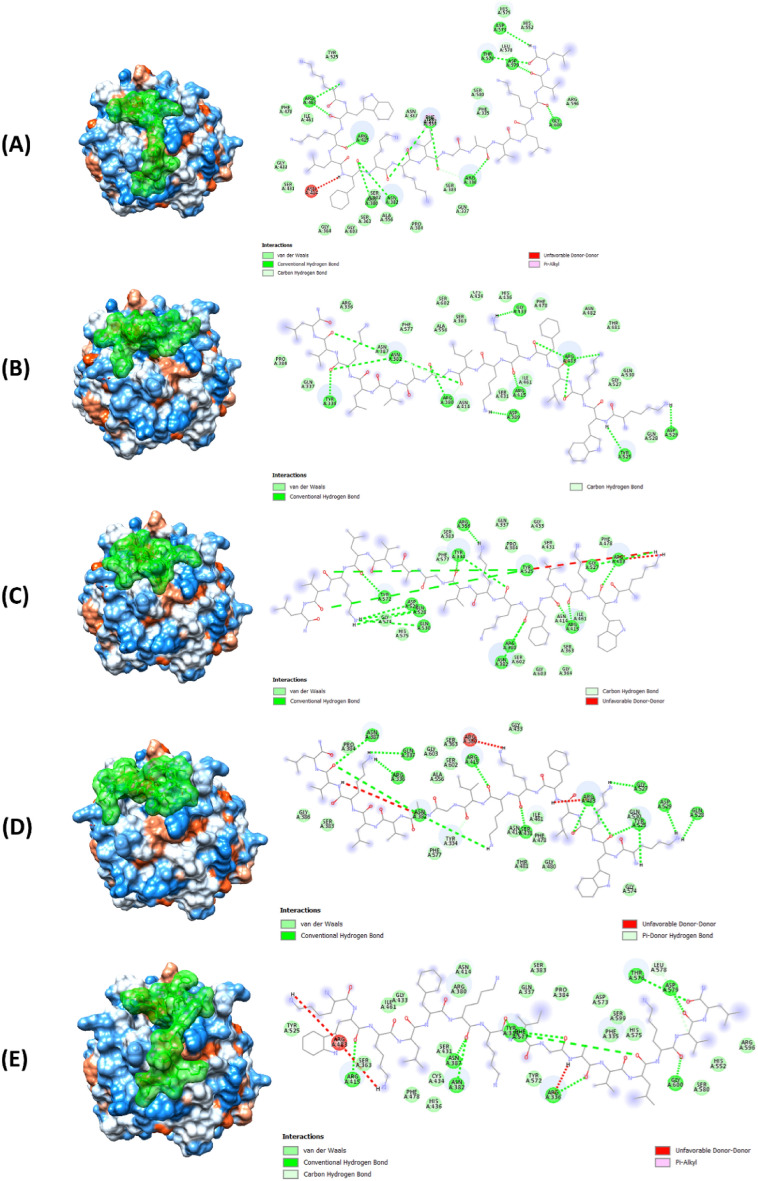
The docking models of melittin (the green molecule) onto Keap1, depicting the interaction patterns observed across multiple docking simulations. **(A, B, C, D, and E)** These figures represented distinct docking patterns, highlighting the diverse binding orientations and interactions between melittin and Keap1. The figure provided insight into the robust interaction profile of melittin with Keap1, showcasing key features such as van der Waals forces, hydrogen bonds, and unique interaction types like pi-donor hydrogen bonds and pi-alkyl interactions. These interactions contributed to the stability and specificity of melittin binding, crucial for its modulatory effects on the Keap1/Nrf2 complex and subsequent release of Nrf2

In the molecular docking analysis, we observed several configurations of intermolecular interactions between melittin and Keap1, each exhibiting the highest predicted affinity of -7.6 kcal/mol across five docking patterns at the Keap1/Nrf2 interaction site (Fig. 9). The presence of diverse interaction types across these patterns suggested compensatory mechanisms that enhance stability and binding efficiency.

Importantly, the strong affinity and stability of the melittin-Keap1 complexes could disrupt or modulate the Keap1-Nrf2 interaction, a critical regulator of cellular responses to oxidative stress.

The interaction density in patterns 2 and 5 (Fig. 9B, E) showed a significantly high number of van der Waals interactions, suggesting extensive surface contacts with Keap1. This is significant, as van der Waals interactions are highly crucial for the stability of protein-ligand complexes. Patterns 1, 3, and 5 (Fig. 9A, C, & E) exhibited a greater number of conventional hydrogen bonds, which are essential for specificity and stability in ligand binding. This indicated that these patterns might form particularly stable complexes with Keap1. The unique pi-donor hydrogen bond in pattern 4 (Fig. 9D) likely facilitated specific and robust binding to certain Keap1 residues.

Additionally, the carbon-hydrogen bonds in patterns 1 and 5 (Fig. 9A, E) and pi-alkyl interactions in these patterns may influence the orientation and stability of melittin within the binding site. While the unfavorable donor-donor interactions noted in patterns 3 and 4 (Fig. 9C, D) might generally be less favorable, their impact is seemingly neutralized by other stabilizing interactions, as evidenced by the consistent docking energy across all patterns. All these properties further suggest a good physical interaction of melittin and Keap1 possible.

**Fig. 10:**
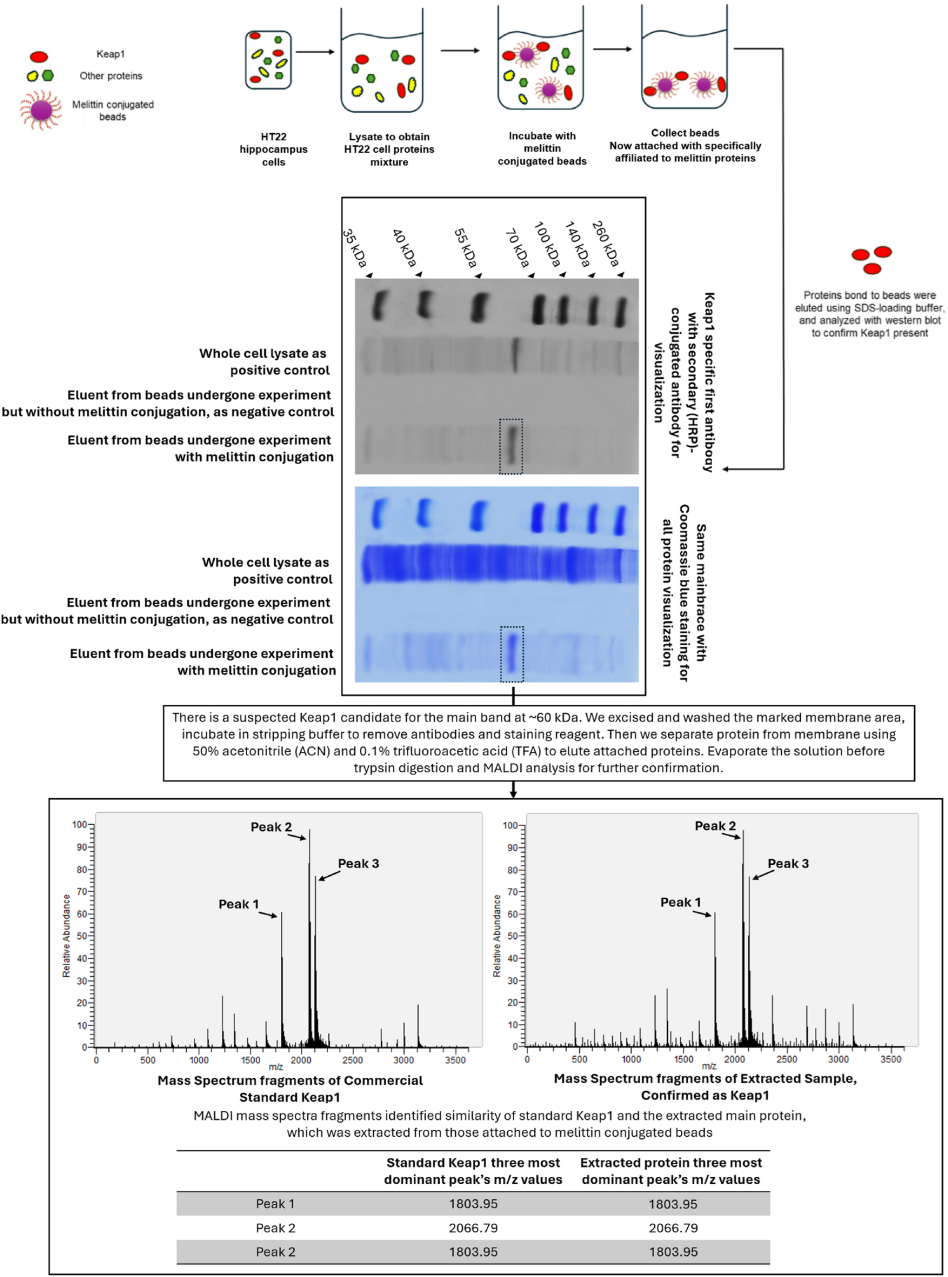
The pull-down assay conducted on HT22 cell lysate to confirm the physical interaction between melittin and Keap1. This assay confirmd the binding of melittin to Keap1 by capturing Keap1 protein complexes using melittin as bait. The presence of Keap1 in the pulled-down samples, extracted from melittin conjugated beads indicated a direct physical association between Keap1 and melittin. Tandem mass spectroscopy also confirmed the main attached protein was Keap1. This experimental validation corroborated the docking simulations and biochemical findings, providing robust evidence for the interaction between melittin and Keap1 at the protein level

Following the 3D docking analysis elucidating the potential interaction between melittin and Keap1, a pull-down assay was conducted to experimentally confirm this interaction. The Keap1 protein is detected approximately at the 60 kDa position. In this assay, melittin was employed as the bait, conjugated on the Epoxy-activated Sepharose 6B beads, to target proteins including Keap1. In the depicted result (Fig. 10), the first lane showed the western blot analysis of the whole HT22 cell lysate, serving as a positive control. The second lane represented a negative control experiment, where the HT22 cell lysate was incubated with beads that were not conjugated with melittin. This lane showed no trace of Keap1, demonstrating that the beads alone do not cause any non-specific binding. This ensured that proteins shown in the third lane are those which specifically bound only to melittin, not bead’s bare surface. The HT22 cell lysate was then incubated with melittin-conjugated beads, which were subsequently collected, washed thoroughly to remove non-specifically bound proteins. SEMS loading buffer, which typically contains sodium dodecyl sulfate (SEMS), a strong anionic detergent, was employed to wash proteins like Keap1 from melittin-conjugated beads and analyzed by western blotting.

The third lane showed the proteins released from the melittin-conjugated beads after washing, with the presence of Keap1 at the 60 kDa, similar to manufacturer description, then stained with Coomassie Brilliant Blue to visualize all proteins. The presence of Keap1 as the main protein bound to melittin beads demonstrated the selectivity of melittin for coupling with Keap1 on a molecular level and thus its potential to activate the downstream Nrf2/HO-1 pathway.

Additionally, mass spectra analysis was conducted to compare the commercial standard Keap1 with the extracted protein sample. The spectra showed three significant peaks, specifically at m/z 1803.95, 2066.79, and 2135.1. These peaks were selected as the most dominant due to their high relative abundance and clear definition in both the commercial standard and the extracted sample spectra. The exact match between the m/z values in the standard and extracted samples reinforced the conclusion that the extracted protein is indeed Keap1. Several peaks appeared exclusively in the extracted sample and not in the standard Keap1 spectra, these peaks are likely attributable to other proteins western blot co-eluted during the extraction process. However, their significantly lower intensity indicates that these proteins presented in much smaller quantities compared to Keap1, and therefore do not represent major components in the sample. This congruence in m/z values supported the hypothesis of specific interaction and binding between melittin and Keap1.

The results of the pull-down assay conclusively demonstrated the physical association between melittin and Keap1, directly supporting the findings from the docking simulations and providing robust evidence for the interaction on a molecular level. This comprehensive approach, combining western blot analysis, Coomassie staining, stripping and re-probing, and mass spectrometry comparison, ensured the specificity and accuracy of Keap1 extraction and identification.

In summary for this invitro section, the study implemented signalling pathway inhibitors, 3D docking models, and pull-down assays to show that melittin activated the Nrf2/HO-1 pathway. Pathway inhibitors highlighted the key role of HO-1. 3D docking showed melittin binding to Keap1, disrupting the Keap1-Nrf2 complex and enabling Nrf2 to activate antioxidant genes. Pull-down assays confirmed this interaction. These findings demonstrated melittin's direct interaction with Keap1, leading to Nrf2 activation.

## Discussion

Our research initially focused on examining melittin's ability to accumulate in the hippocampus tissue, possibly via penetration of the weakened blood-brain barrier (BBB). We utilized the well-established mouse scopolamine-induced neurodegeneration model, commonly used to identify potential anti-neurodegenerative drugs [29–31, 80–82]. In this study, melittin was detected in the hippocampal tissue only in the group pretreated with scopolamine to induce neurodegeneration, and not in normal mice that received melittin alone. BBB is sensitive to stress simulation, in neurodegenerative diseases, the disruption of the BBB allows external elements to infiltrate brain tissues [51–53]. For instance, a weakened BBB permits the passage of large immune cells, such as CD4, to sever in various immune responses, which normally cannot cross this barrier due to their huge size [54]. We believe, normally, an intact BBB filters out melittin and it cannot accumulate much in hippocampus tissue; but in this experiment, scopolamine induced BBB damage enough so that melittin could pass through as shown via the chemical analytical analysis, and this facilitated melittin to interact directly with neurons. Some studies have indicated that BBB disruption is an early reaction in neurodegenerative diseases [80], the recovery process appears to be slow and challenging. This suggests that after melittin administration, there is a critical window during which the damaged BBB allows melittin to enter the brain. This study reveals that melittin selectively penetrates the BBB only in neurodegenerative models, not in healthy controls. This could inform the development of self-adjusted and smart therapeutic strategies for neurodegenerative diseases.

The brain’s high oxygen consumption—approximately 20% of the body's oxygen despite being only 2% of its mass—makes it particularly susceptible to oxidative stress, a central concern in neurodegeneration. Brains also have a considerable amount of lipid content, constituting about 50% of its dry weight. This is essential for neural function, but also makes it prone to lipid peroxidation [24, 81]. Malondialdehyde (MDA), a byproduct of lipid peroxidation, is a critical biomarker of oxidative damage due to its sensitivity and rapid response to oxidative changes. MDA levels rapidly increase following oxidative stress, serving as a real-time indicator of oxidative status, especially in lipid-rich tissues like the brain [23–25, 82]. Similarly, glutathione (GSH), the brain's primary antioxidant, plays an indispensable role in neutralizing ROS and maintaining redox balance. GSH levels can deplete rapidly in response to oxidative stress, making it an early marker of the brain's vulnerability to oxidative damage[23, 27]. The combined assessment of MDA and GSH levels provides a trustworthy indicator of the brain's oxidative stress status.

In previous cardiology and renal studies melittin could help restore cellular redox balance via the Nrf2/HO-1 pathway [20–22]. However, to the best of our knowledge, no research were carried out in vivo level at a neuro-degenerative stage, if melittin can upregulate the diminished Nrf2/HO-1 pathway in animals, and find out why this can happen.

This study is the first to demonstrate that melittin can upregulate the Nrf2/HO-1 pathway in the animal brain tissue, namely the hippocampus. The evidence demonstrated that melittin's initial action primarily involves enhancing HO-1 gene expression, occurring much earlier compared to the subsequent improvements in neurotrophic factors (BDNF and p-CREB), anti-apoptotic protein Bcl-2, inflammation markers (iNOS, TNF-α, IL-6, and IL-1β), and recovery of Ach and ATP levels. Additionally, our in vitro study using the HT22 cell line, along with 3D docking and pulldown assay show strong evidence that melittin can liberate Nrf2 from the Keap1/Nrf2 complex, facilitating Nrf2's translocation into the nucleus and leading to increased HO-1 production, a novel finding.

For a more detail discussion, as we can see the Nrf2/HO-1 pathway showed positive changes before any observed improvements in inflammatory markers (Fig. 7). This indicates the specific interaction mechanisms of melittin prioritized up regulating intracellular antioxidant barrier rather than anti-inflammatory effect. Reduces neuroinflammation is a key strategy in to slow down aging brains [83–86]. Antioxidant and detoxification genes play a crucial role in maintaining cellular homeostasis by eliminating toxins before they cause damage and trigger inflammatory responses. As redox balance is restored, cell regulation improves, leading to a reduction in the overexpression of inflammatory cytokines [3, 15, 79, 87]. In the brain, when inflammation occurs, Microglia cells which have functions similar to macrophage-like cells, and astrocytes which cells offer assistance to neurons, start producing triggering mediators. The activation of both cell types leads to extreme secretion of crucial proinflammatory cytokines such as iNOS, TNF-α, IL-6 and IL-1β. This facilitates negative consequences for neuronal viability and is a signature of inflammation mediated neurodegeneration [88–90]. Therefore, the expressions of TNF-α, iNOS, IL-6 and IL-1β inflammatory substances, were analyzed in this study. But in mice brain, these inflammatory cytokines are mostly suppressed after melittin restored the redox balance in mice brain, indicating a holistic had therapy took place.

A similar pattern was seen in the improvement of brain neurotrophic system signature proteins BDNF and CREB gene expression, which also happened after oxidative damage were suppressed. Normalization of brain neurotrophic indicates a normal working neural cell neuronal cell health and functions. Besides, a strengthen neurotrophic system can also support a more prominent recovery of cellular redox balance. The restoration of this key neurotrophic factor not only improved neuronal cell metabolism but also strengthened the Nrf2-ARE system by activating vibrant neurotrophic downstream signaling pathways, including PI3K/Akt, MAPK (Ras/Ref/Erk), and PLCγ (PKC or CaMK) [79].

This enhancement of intrinsic antioxidant factors led to an overall recovery of neuronal health, positively restoring ATP levels in the tissue, thereby improving many neuronal function [91]. The revival of cellular ATP indicates that the internal metabolic functions of brain cells are returning to normal. In our experiments, we observed that cellular energy recovery lagged behind redox balance restoration, underscoring the detrimental effects of oxidative stress on mitochondrial function which led to decreased ATP production, and cellular survival, as well as its role in initiating apoptosis [92, 93]. These findings provide strong evidence supporting the holistic value of upregulating cellular antioxidant mechanisms in treating neurodegenerative disorders. Melittin treatment reduced Bax levels and increased Bcl-2 levels, indicating an inhibition of apoptosis. Bax promotes apoptosis by permeabilizing the mitochondrial membrane, while Bcl-2 inhibits this process. This recovery is crucial for maintaining neuronal integrity and function, further highlighting melittin's neuroprotective effects [94–96].

These improvements are also believed to contribute to stabilizing cellular signaling matrices, and restoring other neuronal functions, including the cholinergic and neurotransmitter systems, which showed improvement following melittin treatment. These are key targets of well-known anti-dementia drugs such as donepezil [97, 98]. The muscarinic acetylcholine receptor M1 (mAchR1), a subtype of M1-5, is confined to cognitive-related brain regions such as the hippocampus and cortex [99]. It mediates the metabolic action of acetylcholine and increases intracellular calcium concentrations, which activate enzymes related to intracellular signaling systems [61, 100].

To further investigate the precise cellular signaling pathways involved, we conducted in vitro experiments using HT22 mouse hippocampal neurons. This experiment utilized a range of inhibitors to block potential pathways associated with Nrf2/HO-1 activation. These inhibitors help identify the drug's target because when the correct inhibitor is used, it shares the same target as the drug candidate, thus slowing down the pathway that the drug activates. Consequently, the drug becomes less effective, indicating that it was acting on the pathway, which was indicated by the respective inhibitor [37, 58].

The p38, ERK, JNK, and Akt pathways are intricately interconnected in various cellular defense mechanisms, encompassing processes such as inflammation, apoptosis, and cellular regeneration. Importantly, all these pathways can all upregulate the activity of the Nrf2/HO-1 system as stress-response reactions [37, 96, 101–103]. Interestingly, despite their roles, inhibitors of these four pathways fail to replicate the inhibition effect of the HO-1 synthesis inhibitor in effectively blocking melittin's cellular protection which really emphasize melittin's direct influent on the Nrf2/HO-1 activation.

Typically, Nrf2 is held hostage within the cytoplasm by the Keap1/Nrf2 complex. Melittin action, can disguise as a natural defense stimulus to distort this complex’s stability via physical attachments to the Keap1 molecule, which can liberate the intact Nrf2 from the complex and allow it to naturally move into the nucleus, where Nrf2 is the main promoter of important antioxidant gene expression such as HO-1 [73, 76–78].

Molecular docking revealed that melittin binds to Keap1 with multiple high-affinity sites, with binding energies as favorable as −7.6 kcal/mol in the five docking patterns analyzed (Fig. 9). These interactions are predicted to significantly disrupt the Keap1/Nrf2 interaction, thereby facilitating the release of Nrf2 from its inhibitory complex. These results also reveal an interaction profile characterized by a diverse array of van der Waals forces and hydrogen bonds, which are crucial for the stability and specificity of melittin’s binding to Keap1 [82]. The presence of unique interaction types, such as pi-donor hydrogen bonds and pi-alkyl interactions, further substantiates the hypothesis that melittin induces conformational changes in Keap1. These structural alterations are likely to weakened the Keap1-Nrf2 binding, enabling Nrf2 to translocate to the nucleus.

The docking study and pulldown assay results strongly suggest that melittin directly targets Keap1, facilitating the release of Nrf2. This release is a critical initiating event that upregulates the cellular antioxidant defense system, particularly through the activation of the Nrf2/HO-1 pathway [15, 76]. This mechanistic insight aligns well with the observed biochemical and cellular effects in the *in vivo* and *in vitro* experimental results (Figs. 8 and 10), establishing a clear link between melittin's molecular interactions and its neuroprotective efficacy.

Research suggests that disrupting the Keap1/Nrf2 interaction with small molecules enhances the expression of ARE-driven genes like HO-1, which are critical in cellular defense against oxidative stress [76]. This potential makes melittin a promising candidate for therapeutic applications aimed at diseases characterized by oxidative stress and inflammation.

Melittin is typically administered via subcutaneous injection, a method that can cause adverse effects if dosages are excessive. While melittin has shown promise in restoring cognitive function in neurodegenerative models, addressing its potential irritative properties and establishing optimal human dosages are imperative [104]. Recent developments in disease research involving melittin have harnessed recombinant technology and computational bioinformatics to engineer specialized variants with modified amino acid sequences. These innovations have facilitated more effective augmentation and enhanced drug delivery, allowing for intravenous injection and targeted action on specific groups of malaria-infected cells [105]. Such advancements hold the potential to mitigate melittin's side effects and bolster its acceptance as a treatment option.

## Conclusion

In conclusion, our investigation demonstrated for the first time as we know that melittin directly interacts with and reactivates the compromised Nrf2/HO-1 pathway, effectively restoring cellular redox balance. This reactivation establishes a foundation for the natural recovery of various downstream processes, including inflammation, apoptosis, neurotrophic factor regulation, cholinergic function, and mitochondrial performance. At 2,840 Daltons, melittin molecule is huge, and it can have multiple hinden biomolecular interactions from many active sides of its structure for future research. But in this research, under this specific experimental condition, melittin's impact on the Keap1-Nrf2 system appeared to be the most prominent from all the data provided. These findings highlight melittin's potential as a holistic therapeutic agent for conditions marked by oxidative stress and inflammation, supporting the need for further clinical research to explore its therapeutic applications in neurodegenerative diseases.

## Supplementary Information

Additional file 1

## Data Availability

The data that support the findings of this study will be made available upon reasonable request.

## Availability of data and materials

All data generated or analyzed during this study are included in this published article.
