# Supplementary material for: Melittin - the main component of bee venom: a promising therapeutic agent for neuroprotection through keap1/Nrf2/HO-1 pathway activation
Source: Chin Med. 2024 Nov 28;19:166. doi: 10.1186/s13020-024-01020-x (PMC11603938; doi:10.1186/s13020-024-01020-x)
Supplement: Supplementary file 1 — Additional file 1 [file 13020_2024_1020_MOESM1_ESM.docx]

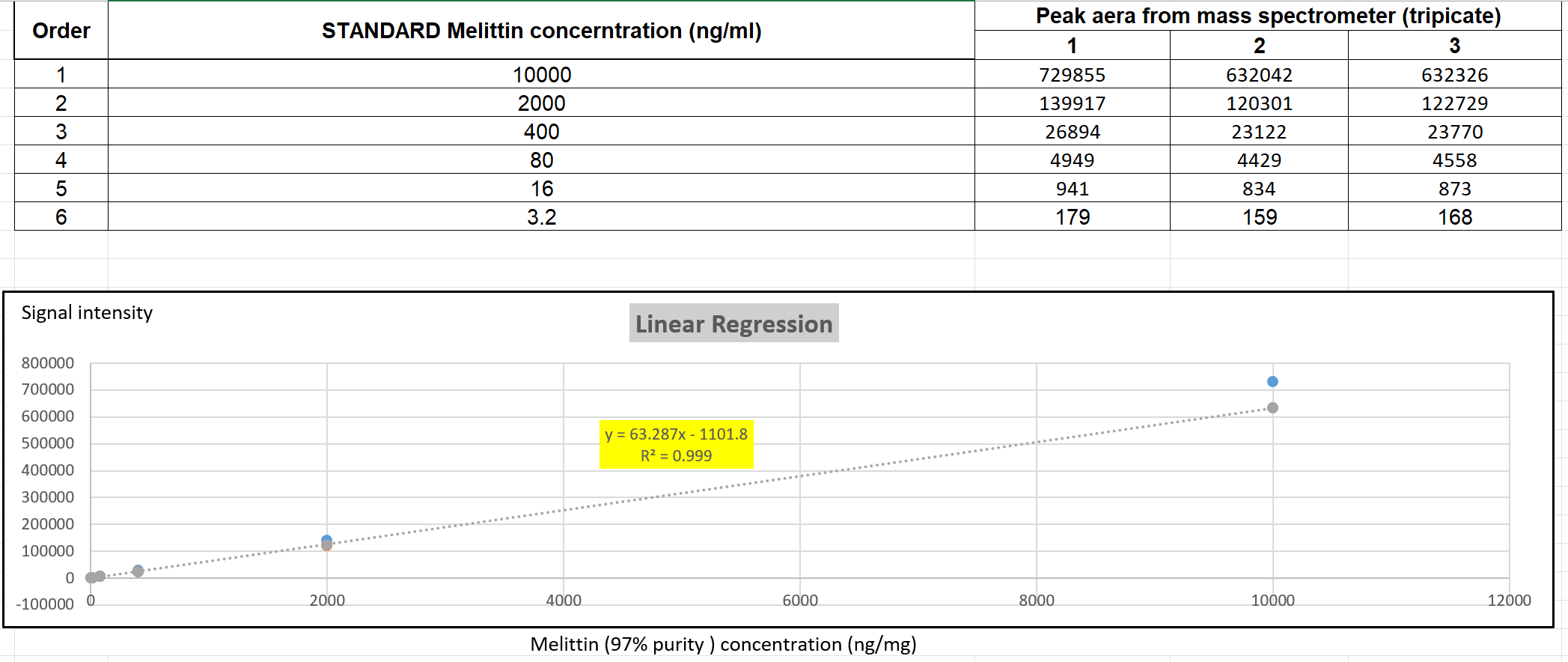


Supplemental figure 1: Formation of Linear regression equation of Melittin, based on quantification of standard Melittin (97% purity)

**Supplemental 1, to section 3.1 in the manuscript: Quantification of Melittin in brain tissue sample**

This supplemental data represents the quantification of Melittin in brain tissue samples using mass spectrometry. Section 3.1 with Fig 3 already explain how to identify melittin with specific m/z values of main fragments; and how to identify melittin peak (at m/z 712.44) from all sample chromatogram at Rt~7.04 min. This supplement explains further how the values of melittin concertation in brain samples were calculated:

**1, Standard Curve Creation**: The upper table of Supplemental figure 1 displays the signal intensities obtained from mass spectrometer readings in triplicate for varying concentrations of standard Melittin ranging from 10 ng/mg to 0.0032 ng/mg that were prepared and injected to the LC-MS/S system.

**2, Linear Regression and Calibration**: The lower chart depicts the corresponding of linear regression analysis, which plots the injected Melittin concentration against the signal intensity. The resulting linear y = 63.287x - 1101.8 with R² = 0.999.

with an R^2^ =0.999demonstrates a strong linear relationship, indicating that the mass spectrometer readings accurately reflect the concentrations of Melittin in the brain tissue samples.

This regression equation is used to determine the Melittin concentrations in experimental samples by correlating their signal intensities with the standard curve generated from these known concentrations of Melittin. This method provides precise quantification, critical for assessing the distribution and bioavailability of Melittin in the brain after systemic administration.


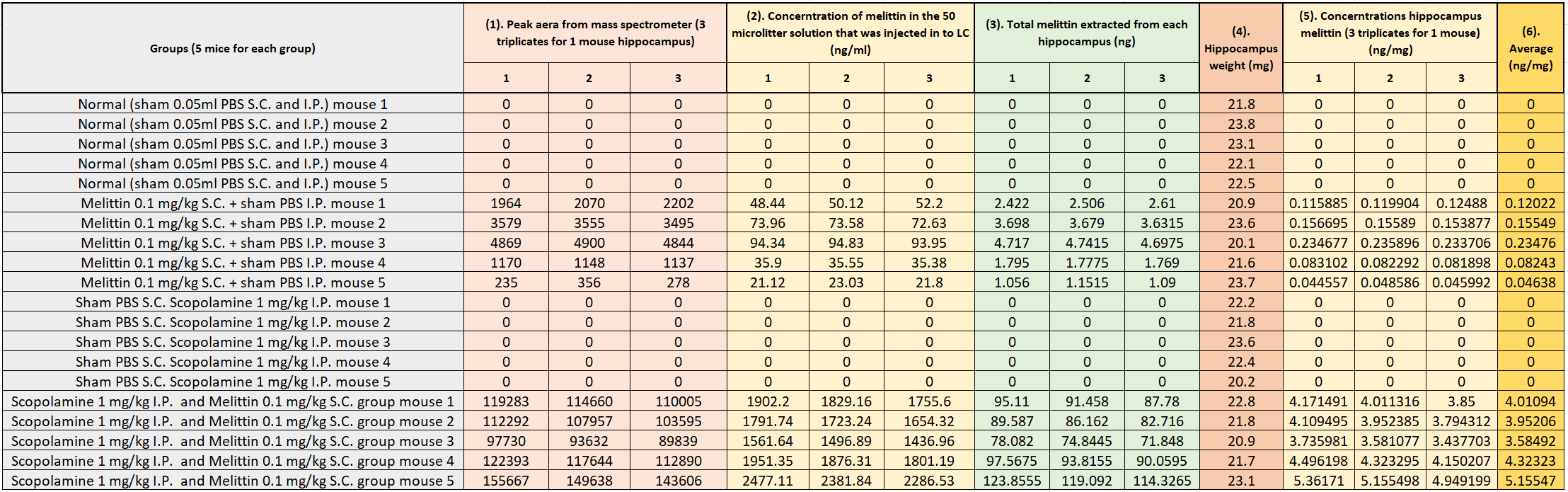


**3, Cross-Verification**: This table presents the quantification results of Melittin in brain tissue samples from different experimental groups, as measured by mass spectrometry. The groups include normal control mice, mice treated with melittin alone, mice treated with scopolamine alone, and those treated with both scopolamine and melittin. The data includes the signal intensity readings from the mass spectrometer (measured in triplicate for each mouse) and the corresponding calculated Melittin concentrations (ng/mg of tissue).

- Normal (Sham PBS) Group: No Melittin was detected across all samples, as expected.
- Melittin only Group: Mice treated with 0.1 mg/kg Melittin exhibited low but detectable levels of Melittin in brain tissue, with concentrations ranging from approximately 0.022 to 0.235 ng/mg, depending on the individual mouse.
- Scopolamine only Group: No Melittin was detected across all samples, as expected.
- Scopolamine + Melittin Group: Mice treated with both scopolamine and Melittin showed significantly higher levels of Melittin in brain tissue, with concentrations averaging between 3.58 and 5.15 ng/mg.

The average concentration values, calculated from the triplicate measurements, indicate a consistent presence of Melittin in brain tissues following administration. This data demonstrates the successful delivery and quantification of Melittin, as well as its enhanced accumulation in the brain when co-administered with scopolamine.

Supplemental figure 2: Description of Quantification Results

**3, Cross-Verification for melittin concentration if hippocampus from Linear Regression and Calibration**:

The right hippocampus from each mouse (5 per group) was lysed and centrifuged at 2500g. The aqueous supernatant, which contains water-soluble melittin, was collected and freeze-dried. The dried material was then dissolved in 50 microliters of pure water, filtered into a vial, and subjected to analysis with a 5-microliter injection into the LC system.

Formula for each result above:

**(1):** Were collected from mass spectrometer data, 3 injections for 3 repeated runs were carried out from prepared 1 vial.

**(2)**= (**(1)+**1101.8)/63.287; Convert from mass signal to concentration based on the formulated Linear Regression equation.

**(3)**=**(2)***0.05; Weight=Concentration*Volume.

**(4)**: Hippocampus weight measured after collection from brain, before melittin extraction.

**(5)**=**(3)/(4)**; Concentration=Weight of melittin/weight of hippocampus.

**(6)**= Average of 3 triplicates of **(5)**.

The average concentration values, calculated from the triplicate measurements, indicate a consistent presence of Melittin in brain tissues following administration. Especially, this data demonstrates the successful delivery and quantification of Melittin, as well as its enhanced accumulation in the brain when co-administered with scopolamine.

Supplemental figure 3: Full version of behaviour data, explained the involvement of

an extract group treated with Donepezil and served as positive control group

This supplemental figure highlights the behavioural effects of Donepezil in a scopolamine-induced cognitive impairment model, compared to Melittin and control groups.

**(A) Escape Latency in the Morris Water Maze (MWM):** Donepezil significantly reduced escape latency, demonstrating its effectiveness in improving cognitive function in scopolamine-impaired mice. Mice treated with Donepezil reached the platform more quickly over successive days, indicating enhanced spatial learning and memory. The Melittin-treated group also showed a reduction in escape times, though the effect was slightly less pronounced than with Donepezil.

**(B) Spatial Memory Performance Heat Maps:** Heat maps display that Donepezil-treated mice exhibited focused search patterns in the target quadrant during the MWM test, indicating strong memory retention. Melittin-treated mice also improved in spatial memory, but their search patterns were broader and less concentrated compared to those treated with Donepezil.

**(C) Spontaneous Alternation in the Y-Maze:** Donepezil effectively restored spontaneous alternation percentages in the Y-Maze, a measure of working memory, bringing it closer to normal control levels. This reflects its protective effects on cognitive function. Melittin also enhanced spontaneous alternation, showing some improvement in working memory, though not to the same extent as Donepezil.

Donepezil restored spontaneous alternation in the Y-Maze, reflecting improved working memory, while Melittin showed moderate improvement.

Overall, the data confirms Donepezil as an effective positive control in reversing cognitive deficits, and it also suggests Melittin’s potential neuroprotective effects.


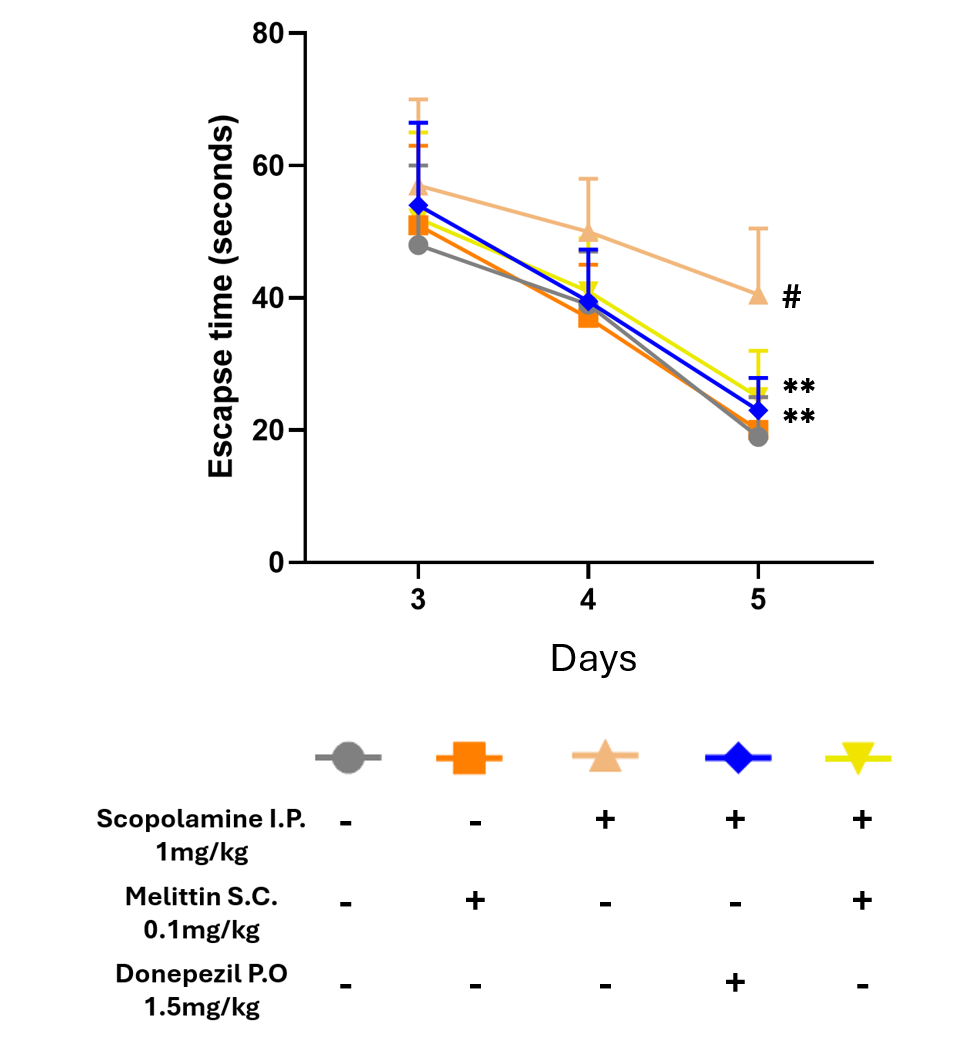

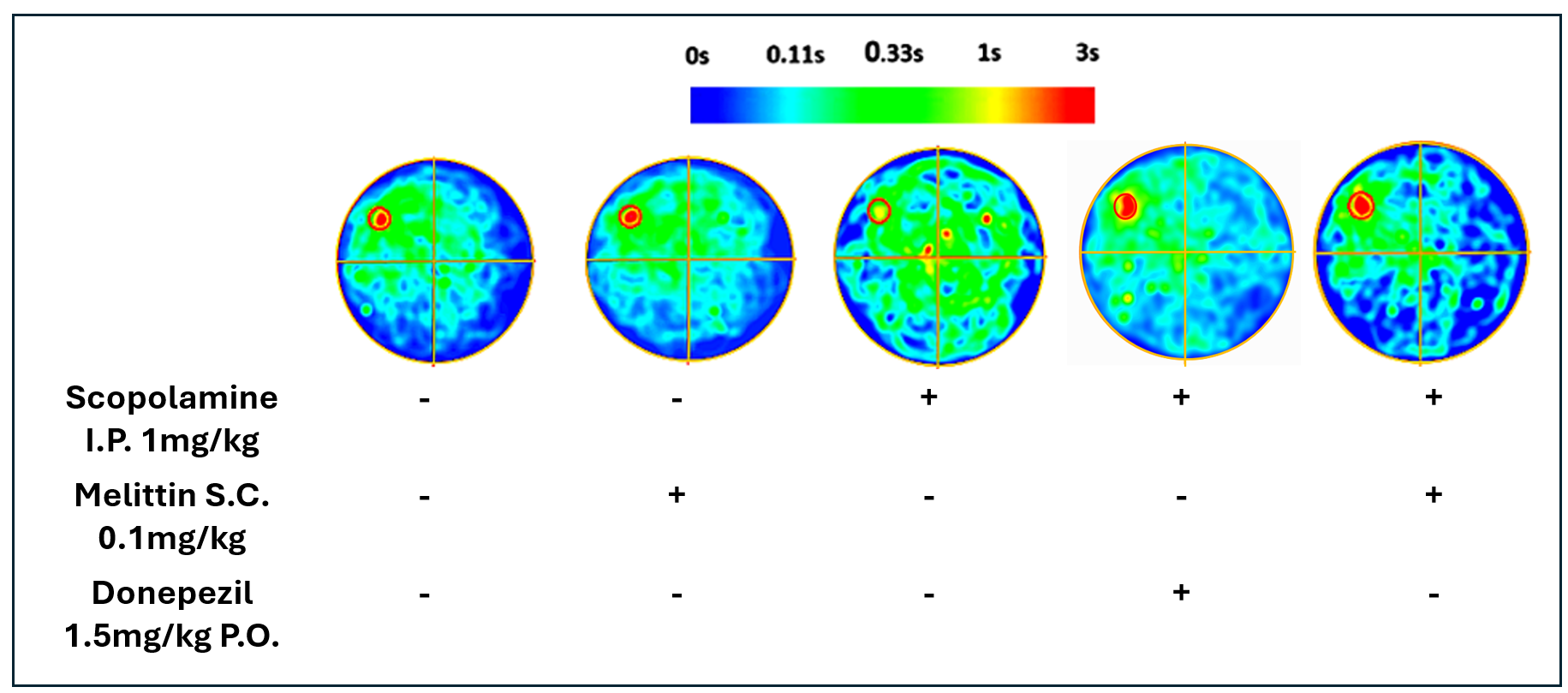

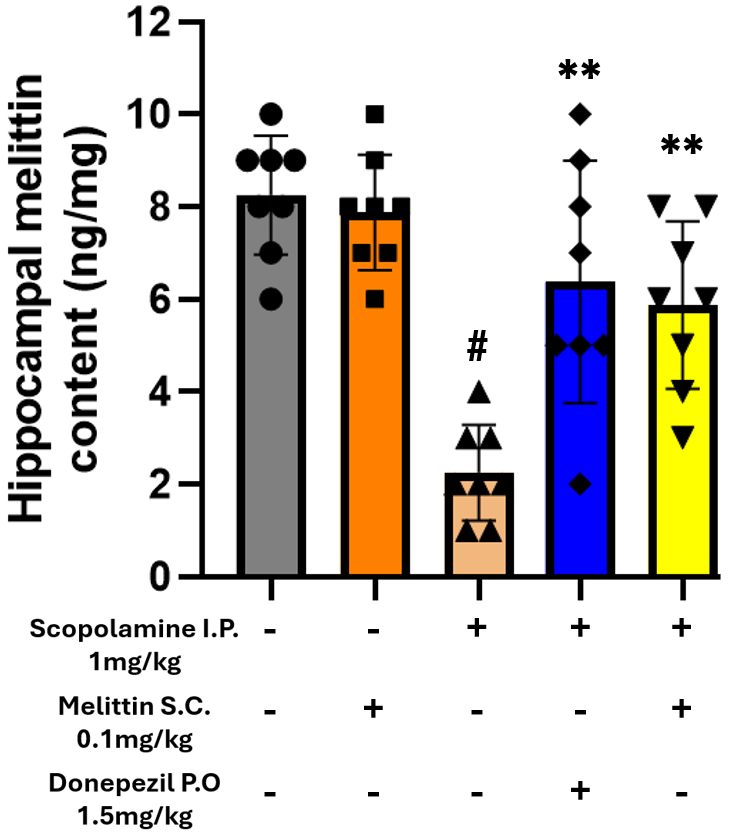

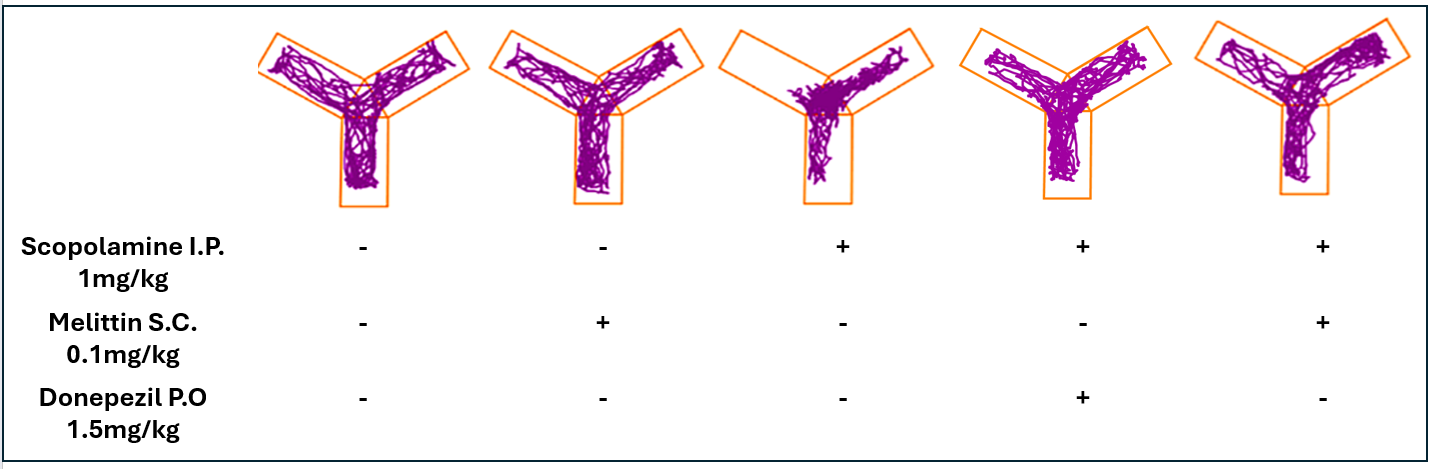

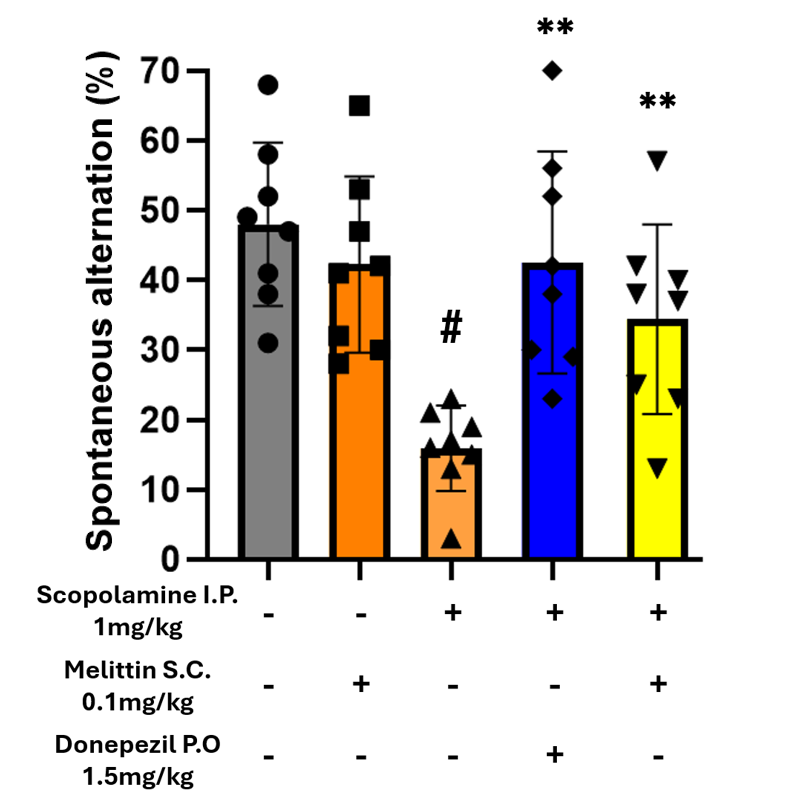

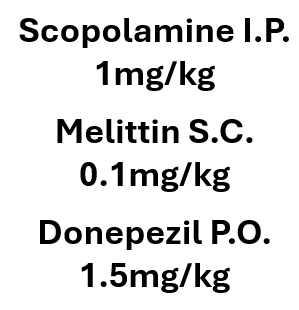

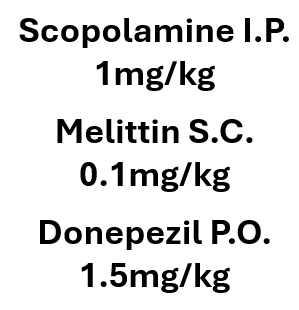

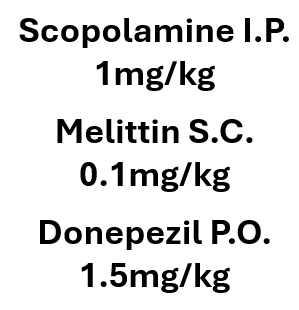


**Supplemental 2, to section 3.2 The data of the positive control with Donepezil per os (P.O.) 1.5 mg/kg**

**(A)**

**(A)**

**(C)**

**(B)**
